# An in vitro platform for engineering and harnessing modular polyketide synthases

**DOI:** 10.1038/s41467-019-13811-0

**Published:** 2020-01-03

**Authors:** Takeshi Miyazawa, Melissa Hirsch, Zhicheng Zhang, Adrian T. Keatinge-Clay

**Affiliations:** 1 Department of Molecular Biosciences, The University of Texas at Austin, 100 E. 24th St., Austin, TX 78712 USA; 2 Department of Chemistry, The University of Texas at Austin, 100 E. 24th St., Austin, TX 78712 USA

**Keywords:** Biocatalysis, Metabolic engineering, Multienzyme complexes

## Abstract

To harness the synthetic power of modular polyketide synthases (PKSs), many aspects of their biochemistry must be elucidated. A robust platform to study these megadalton assembly lines has not yet been described. Here, we in vitro reconstitute the venemycin PKS, a short assembly line that generates an aromatic product. Incubating its polypeptides, VemG and VemH, with 3,5-dihydroxybenzoic acid, ATP, malonate, coenzyme A, and the malonyl-CoA ligase MatB, venemycin production can be monitored by HPLC and NMR. Multi-milligram quantities of venemycin are isolable from dialysis-based reactors without chromatography, and the enzymes can be recycled. Assembly line engineering is performed using pikromycin modules, with synthases designed using the updated module boundaries outperforming those using the traditional module boundaries by over an order of magnitude. Using combinations of VemG, VemH, and their engineered derivatives, as well as the alternate starter unit 3-hydroxybenzoic acid, a combinatorial library of six polyketide products is readily accessed.

## Abstract (web summary)

A robust platform to study modular polyketide synthases (PKSs) in vitro is still unavailable. Here, the authors report the reconstitution of the venemycin PKS, engineer hybrid venemycin/pikromycin PKSs, and obtain much improved yields through employing the updated module boundaries.

## Introduction

Modular polyketide synthases (PKSs) are among the most miniature assembly lines known, yet they produce some of the most important human medicines, such as the antibacterial erythromycin and the anticancer agent epothilone^1,2^. While synthetic chemists and material scientists alike yearn to reprogram these carbon–carbon bond forming, stereocenter-setting machines to produce new medicines and polymers, they can be challenging to work with, as they are comprised of several, difficult-to-purify polypeptides encoded by large, GC-rich genes. The only reconstituted PKS assembly line that has been reported is the erythromycin synthase; however, its first polypeptide was expressed in three pieces, and the product of the assembly line was only detectable by mass spectrometry^3^.

**Fig. 1:**
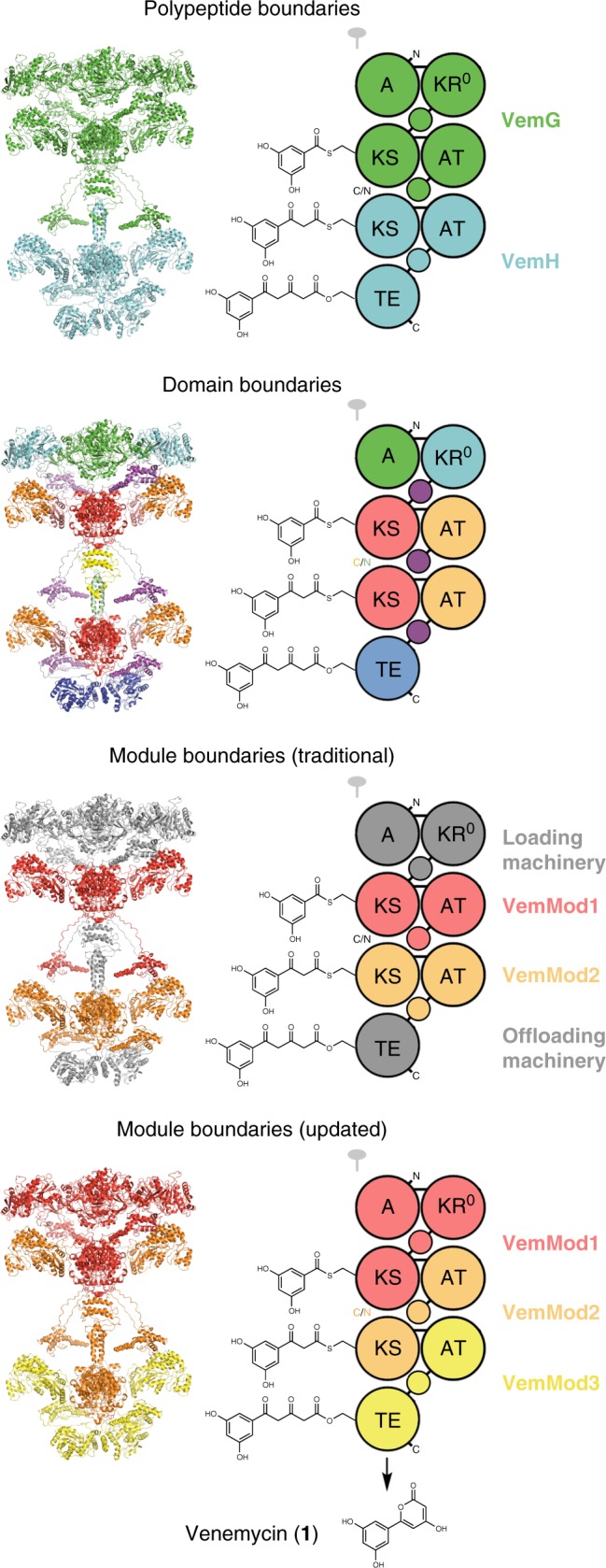
The venemycin synthase viewed from several perspectives. Its polypeptides, the 232 kDa VemG and the 140 kDa VemH, are threaded through known structures to create an all-atom model of this short polyketide assembly line. An adenylation (A) domain accepts a 3,5-dihydroxybenzoyl starter unit, and malonyl groups, transferred from malonyl-CoA to acyl carrier protein (ACP) domains (small circles) by acyltransferases (ATs), extend the growing chain within the active sites of ketosynthases (KSs). A thioesterase (TE) catalyzes cyclization to yield the aromatic product venemycin. C- and N-terminal docking domains are represented by C/N. An inactive ketoreductase (KR^0^) may play a structural role. The flanking subdomains (also known as KS/AT adapters in *cis*-AT PKSs), which are C-terminal to KS domains, appear pink in the model but are not depicted in the cartoon. Traditional module boundaries run from the N-terminal end of KS domains to the C-terminal end of ACP domains. Recently, module boundaries were redefined at the C-terminal end of KS domains to reflect the evolutionary co-migration of and collaboration between assembly line domains.

We in vitro reconstitute the three-module venemycin synthase (the term module reflects the updated definition throughout this report, unless otherwise stated) (Fig. 1)^4–6^. As one of the shortest polyketide assembly lines (some contain >30 modules) and as the producer of an easily detectable product, it provides a tractable platform and a foothold to learn more about these molecule factories. Since few successes in engineering assembly lines with the traditional boundaries have been reported, our lab is particularly interested in testing a recent hypothesis concerning the boundaries of modules: bioinformatic investigation of related aminopolyol-producing synthases revealed that the set of domains that genetically co-migrates during the evolution of polyketide assembly lines differs from the traditional definition of a module and suggested that the downstream end of a module should include the KS traditionally considered to be part of the subsequent module^5–7^. We show that synthases engineered using the updated boundaries are over an order of magnitude more active than those engineered using the traditional boundaries^8–11^.

## Results

### In vitro reconstitution of the venemycin assembly line

**Fig. 2:**
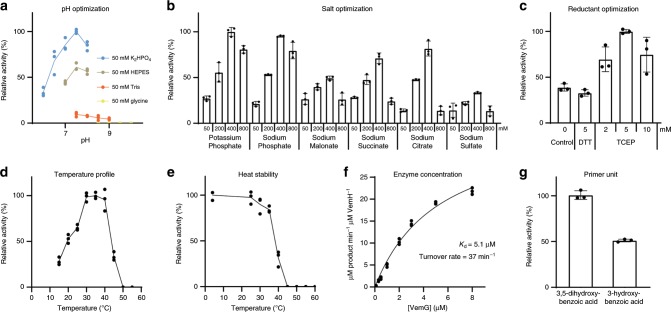
In vitro reconstitution and reaction optimization of the venemycin assembly line. **a** The pH activity profile indicates pH 7.5 is optimal. **b** In a salt screen, 400 mM potassium phosphate is best. **c** TCEP is better than DTT as a reductant. **d** Reactions are fastest at 30–35 °C. **e** However, the enzymes are not stable at these temperatures - activity after incubation of VemG and VemH at 4–55 °C for 30 min is shown. **f** Measuring activity at increasing concentrations of VemG (at constant 0.2 µM VemH), enables the determination of the turnover rate and an apparent *K*_d_. **g** The assembly line accepts 3-hydroxybenzoic acid but is twofold slower. Data are mean ± s.d., *n* = 3 independent experiments. Source data are provided as a Source Data file.

The genes encoding VemG and VemH were cloned from *Streptomyces venezuelae* ATCC 10712 genomic DNA into expression vectors such that the histidine tags used in the purification are positioned at the assembly line termini, distant from the docking domain interface formed by the C- and N-terminal ends of VemG and VemH, respectively (Supplementary Figs. 1 and 2 and Supplementary Table 1)^10,12^. The acyl carrier protein (ACP) domains of the synthase are phosphopantetheinylated by the promiscuous enzyme Sfp (engineered into the *Escherichia coli* K207-3 expression host) and are thus functionalized to relay the growing chain in a hand-over-hand manner through the assembly line^13,14^. Extender units are supplied by a malonyl-CoA regeneration system comprised of *Streptomyces coelicolor* MatB, malonate, CoA, and ATP^15^. Through optimizing the reaction conditions (400 mM potassium phosphate, 5 mM TCEP, pH 7.5 at 25 °C) and the concentrations of the enzymes (8 µM VemG, 8 µM VemH, and 10 µM MatB) and substrates (0.75 mM 3,5-dihydroxybenzoic acid, 10 mM malonate, 1 mM CoA, 9 mM ATP) 100 µL reactions went to completion in 2 h (turnover rate = 37 min^−1^) (Fig. 2 and Source Data). The apparent *K*_d_ for the VemG/VemH interaction was measured to be 5.1 µM, over an order of magnitude stronger than the affinity measured between the docking domains alone^16^. When the concentrations of VemG and VemH are much below this value, the speed of the reaction is essentially governed by a second-order rate constant and considerably slower. The reconstituted assembly line can incorporate 3-hydroxybenzoic acid, albeit at half the rate of the natural 3,5-dihydroxybenoic acid starter unit.

**Fig. 3:**
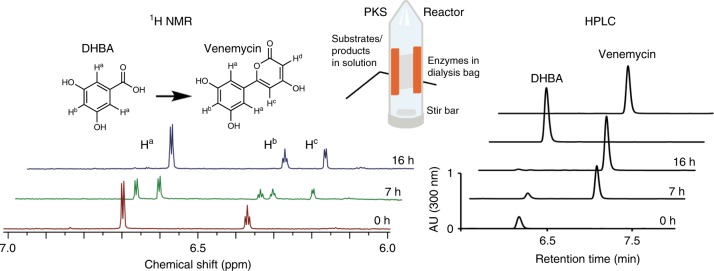
The PKS reactor facilitates scaled-up reactions. The 100 kDa MWCO dialysis bag ensures high concentrations of the assembly line polypeptides, facilitates analysis of the reaction by NMR and HPLC, and enables transfer of the enzymes to subsequent reactors (half-life: 2 days).

Scaled-up reactions were performed in 45 mL reactors, in which a dialysis bag maintains a high enzyme concentration and reaction progress can be monitored by HPLC or NMR analysis of the surrounding solution (Fig. 3). In a typical reaction, the 3,5-dihydroxybenzoic acid starter unit is quantitatively converted into ~8 mg of venemycin (**1**) over 16 h. The polyketide product can be rapidly isolated without chromatography and characterized (e.g., NMR, x-ray crystallography), and the enzymes can be recycled in subsequent reactors with a half-life of 2 days.

### Hybrid assembly lines

**Fig. 4:**
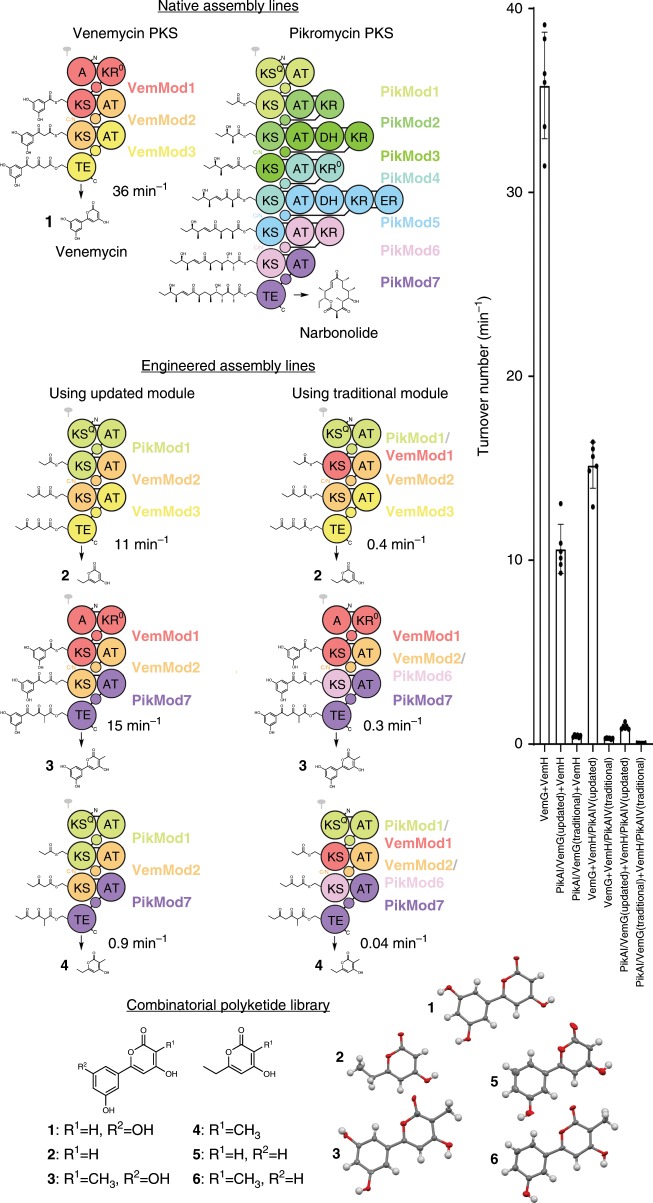
Engineering and harnessing polyketide assembly lines. The venemycin and pikromycin synthases are combined to yield several hybrids. Assembly lines designed using the updated module boundaries outperform those using the traditional module boundaries by over an order of magnitude (25- and 48-fold, data are mean ± s.d., *n* = 6 independent experiments). A combinatorial library of 6 products (**1**–**6**) is accessed through the engineered synthases and through the alternative starter unit, 3-hydroxybenzoic acid (crystal structures shown in thermal elipsoids). Source data are provided as a Source Data file.

Using the updated module definition, a hybrid synthase comprised of the first module from the pikromycin assembly line (*S. venezuelae* ATCC 15439) and the second and third modules from the venemycin assembly line was designed (Fig. 4 and Supplementary Data 1)^17^. To generate the desired PikAI/VemG construct, amplicons containing portions of *pikAI* and *vemG* were joined through the Seamless Ligation Cloning Extract (SLiCE) method^18^. Purified PikAI/VemG and VemH were entered into a reactor in which both malonyl- and methylmalonyl-CoA are regenerated by MatB, as the pikromycin loading module makes a propionyl starter unit through the decarboxylation of a methylmalonyl group obtained from methylmalonyl-CoA. As MatB prefers malonate to methylmalonate, 1.6 mM malonate and 8.4 mM methylmalonate were supplied^19^. The anticipated pyrone (**2**) was produced quantitatively (11 min^−1^). A hybrid synthase comprised of the first two modules of the venemycin assembly line and the last module of the pikromycin assembly line (VemG and VemH/PikAIV) produced the anticipated methyl-substituted venemycin (**3**) quantitatively (15 min^−1^). Combining PikAI/VemG and VemH/PikAIV also yielded the anticipated methyl-substituted pyrone (**4**), albeit at a slower rate (0.93 min^−1^).

Analogous hybrid synthases were also designed using the traditional module boundaries, with the junctions between PikAI and VemG as well as VemH and PikAIV immediately N-terminal to the KS domains. Synthases composed of PikAI/VemG and VemH as well as VemG and VemH/PikAIV were significantly less active than the hybrid synthases designed using the updated module boundaries (25- and 48-fold, respectively). The synthase composed of both PikAI/VemG and VemH/PikAIV was also less active than the synthase constructed with updated module boundaries (27-fold).

### Combinatorial polyketide library

Compared to other classes of natural products, a larger proportion of polyketides possess medicinally-relevant bioactivities, such as cytotoxicity to cancer cells, and have been developed into medicines^20,21^. However, the medicinal chemistry involved in exploring variations of such stereochemically rich compounds can be prohibitively challenging. By collaborating with the assembly lines that naturally biosynthesize these molecules, combinatorial libraries of polyketide drug leads may be accessable. As proof of this concept, a combinatorial library of six compounds was biocatalytically generated using modules from the venemycin and pikromycin assembly lines and the alternative starter unit 3-hydroxybenzoic acid (Fig. 4). The synthesis was environmentally friendly, inexpensive, rapid, conducted in one pot, and free of chromatography. Characterization, including crystal structures for five of the six compounds, was facile (Supplementary Methods and Supplementary Figs. 3–31).

## Discussion

The lower activities of synthases engineered using the traditional module boundaries may be due to weaker interactions between ACPs and downstream KSs that did not evolutionarily co-migrate. Previous studies have shown that maintaining ACPs with the KSs naturally C-terminal to them in an engineered assembly line can be beneficial^22,23^. This domain connection is quite clear in the related *trans*-acyltransferase (AT) assembly lines (in which the AT domains that charge ACPs with extender units reside on separate polypeptides, in contrast to *cis*-AT assembly lines like the pikromycin and venemycin synthases) through bioinformatics^7^.

The junction between modules in *trans*-AT assembly lines is localized on a LPTYPFxxxxxW motif C-terminal to the KS flanking subdomain^24^ (Fig. 1) and is easy to observe in sequence alignments owing to the diverse module types in these synthases^7^. In contrast, the junction between the modules of *cis*-AT assembly lines is delocalized from the N-terminal end of the KS domain to the C-terminal end of the AT domain. *cis*-AT assembly lines are almost entirely composed of a few variations of a single module type - modules that either possess a full complement of processing enzymes [ketoreductase (KR), dehydratase (DH), enoylreductase (ER)] or smaller versions that contain fewer or none of these processing enzymes. Having essentially one module type may confer an evolutionary advantage to *cis*-AT assembly lines in that productive recombination events can occur in more locations, as observed in variants of the rapamycin synthase in which junctions occurred internal to KS and AT domains^25^. Even when recombination in a *cis*-AT assembly line separates an ACP from the enzymes of its module the recombined synthase may still be functional, as ACPs from different *cis*-AT modules are quite homogenous, especially in comparison to ACPs from different *trans*-AT module types. Our group chose a recombination site N-terminal to the flanking subdomain connecting the KS and AT domains so that its structural integrity would not be jeopardized^24,26^.

Many facets of assembly line biochemistry can be studied with the described platform, such as enzyme specificities, processivity versus iteration, ACP docking, unstructured linkers, and docking domains. The structural biology of assembly line enzymes also merits deeper investigation^26–28^. We generated an all-atom model of the venemycin synthase to aid in these structure/function studies (Supplementary Fig. 1), although a crystal structure or electron microscope reconstruction of a short assembly line would be ideal. Assembly line engineers can utilize reactors to monitor the behavior of designed synthases in vitro and trouble-shoot before their employment in vivo. Shunt products isolated from suboptimal synthases can be quickly characterized to guide improved designs. We envision engineering short assembly lines containing processing enzymes and even modules from *trans*-AT and nonribosomal peptide synthetase (NRPS) assembly lines to produce diverse, stereocomplex products. The described platform should significantly aid efforts to construct larger assembly lines that synthesize bioactive molecules and, ultimately, new medicines.

## Methods

### Reagents and equipment

The KAPA HiFi DNA polymerase is from KAPA Biosystems. All restriction enzymes are from New England Biolabs. All primers were custom ordered from Sigma-Aldrich. Luria-Bertani (LB) Miller Broth, sodium phosphate, potassium phosphate, sodium chloride, glycerol, and HEPES are from Fisher Scientific. Kanamycin sulfate, dithiothreitol (DTT), and sodium citrate dihydrate are from VWR. Carbenicillin is from Amersham. Isopropyl-β-d-thiogalactopyranoside (IPTG) is from Carbosynth. Ni-NTA affinity resin and tris(2-carboxyethyl)phosphine hydrochloride (TCEP-HCl) are from Thermo Fisher Scientific. MonoQ 5/50, Superdex 200 Increase 10/300, Sephacryl S-300 HR, HiLoad 16/600 Superdex 200 pg, and Superdex 200 Increase 16/600 GL are from GE Healthcare. Magnesium chloride is from MACRON. Tris(hydroxymethyl)aminomethane is from MP Biomedicals. Malonic acid and methylmalonic acid are from Oakwood Chemical. 3,5-Hydroxybenzoic acid, magnesium sulfate, sodium succinate, 4-hydroxy-6-methyl-2-pyrone, adenosine triphosphate (ATP), imidazole, and glycine are from Sigma-Aldrich. Coenzyme A (CoA) is from Oriental Yeast Co., Ltd. 3-Hydroxybenzoic acid is from Alfa Aesar. Amicon Ultracentrifugal protein concentrators filters are from Millipore. Ethyl acetate (EtOAc), acetonitrile, methanol, chloroform, and hexanes are from Fisher Scientific. D_2_O, CDCl_3_, and DMSO-d_6_ are from Cambridge Isotope Laboratories. SiliaFlash Irregular Silica Gels are from SiliCycle.

FPLC chromatography was performed using a Biologic DualFlow instrument (Bio-Rad). HPLC analysis was carried out on a Waters 1525 HPLC system. High-resolution mass spectral analysis was conducted on a 6230 TOF LC/MS (Agilent Technologies). ^1^H and ^13^C NMR spectra were collected on a Bruker AVIII HD 400 (400 MHz) supported by Grant Willson and Benjamin Keitz (CHE-1626211) and Bruker AVIII HD 500 (500 MHz) supported by Jonathan Sessler (NIH 1 S10 OD021508-01).

*E. coli* DH5α was used for plasmid construction. BL21(DE3)star pLysS cells were used for MatB expression. *E.coli* K207-3 cells were used for the expression of assembly line enzymes^13^.

### Construction of protein expression plasmids

To construct expression plasmids for the venemycin assembly line enzymes, the DNA encoding VemG and VemH was PCR amplified from genomic DNA of *Streptomyces venezuelae* ATCC 10712. The resulting *vemG* and *vemH* amplicons were SLiCE assembled into NdeI/XhoI sites of pET28b-His10 (constructed from pET28b through SLiCE using primers in Supplementary Table 1) and pET21a, respectively^18^. To construct PikAI/VemG fusion protein expression plasmids, PikAI portions and VemG portions were obtained by PCR amplification with genomic DNA of *Streptomyces venezuelae* ATCC 15439 and pET28-VemG, respectively, and SLiCE assembled into NdeI/XhoI sites of pET21a. To construct VemH/PikAIV fusion protein expression plasmids, the VemH and PikAIV portions were obtained by PCR from pET21-VemH and genomic DNA of *Streptomyces venezuelae* ATCC 15493, respectively and SLiCE assembled into NdeI/XhoI sites of pET21a. All primers used for plasmid construction as well as protein sequences are reported (Supplementary Table 1 & Supplementary Data 1).

### Protein expression and purification

All assembly line enzymes were heterologously expressed in *E. coli* K207-3 cells, while MatB was expressed in *E. coli* BL21(DE3) star (pLysS) cells. The *E. coli* transformed with the expression plasmid were grown on LB medium containing 50 mg mL^−1^ kanamycin to OD_600_ = 0.6 at 37 °C, shaking at 225 rpm. Cultures were induced through the addition of 0.5 mM IPTG and then incubated for 18 h at 15 °C. Cells were harvested by centrifugation (4000 × *g* for 20 min), resuspended in lysis buffer (50 mM potassium phosphate, 500 mM NaCl, 5 mM imidazole, 1 mM TCEP, 10% v/v glycerol, pH 7.5), sonicated, and the cell debris was removed by centrifugation (20,000 × *g* for 30 min). The supernatant was applied to a Ni-NTA column (2 × 4 cm) and washed with 5 column volumes of lysis buffer containing 15 mM imidazole. The His-tagged protein was eluted with 2 column volumes of lysis buffer containing 250 mM imidazole. His-tagged MatB was concentrated using an Amicon Ultra centrifugal filter (Merck), and the buffer was exchanged for storage buffer (50 mM HEPES, 150 mM NaCl, 10% v/v glycerol, pH 7.5). His-tagged VemG was further purified using a Superdex 200 Increase 10/300 GL column equilibrated with size exclusion buffer (50 mM potassium phosphate, 150 mM NaCl, 1 mM TCEP, 10% v/v glycerol, pH 7.5). His-tagged PikAI/VemG chimeric proteins were further purified using a HiLoad Superdex 16/600 200 pg column (GE Healthcare) equilibrated with size exclusion buffer. His-tagged VemH and VemH/PikAIV chimeric proteins were applied to a MonoQ 5/50 column equilibrated with 95% Buffer A (50 mM potassium phosphate, 1 mM TCEP, 10% v/v glycerol, pH 7.5) and 5% Buffer B (50 mM potassium phosphate, 1 M NaCl, 1 mM TCEP, 10% v/v glycerol, pH 7.5). The proteins were eluted with a linear gradient of 5–50% Buffer B. Final purification was performed with a Superdex 200 Increase 10/300 GL column equilibrated with the size exclusion buffer. Protein concentrations were estimated using the *ε*_280_ values calculated by ExPASY ProtParam (https://web.expasy.org/protparam/): *ε*_280_ (His_10_ + VemG) = 156,760 M^−1^ cm^−1^, *ε*_280_(VemH + His_6_) = 125,820 M^−1^ cm^−1^, *ε*_280_(VemH/PikAIV(updated) + His_6_) = 150,800 M^−1^ cm^−1^, *ε*_280_(VemH/PikAIV(traditional) + His_6_) = 152,290 M^−1^ cm^−1^, *ε*_280_(His6 + PikAI/VemG(updated)) = 292,740 M^−1^ cm^−1^, *ε*_280_(His_6_ + PikAI/VemG(traditional)) = 292,740 M^−1^ cm^−1^, and *ε*_280_(His_6_ + MatB) = 33,920 M^−1^ cm^−1^. Protein quality was assessed by SDS-PAGE analysis and size-exclusion chromatography (Supplementary Fig. 2). VemG and PikAI/VemG hybrids apparently oligomerize, eluting in the void volume even on a Sephacryl S-300 HR column that has a 0.01–1.5 MDa fractionation range. VemH and VemH/PikAIV hybrids clearly elute after the void volume on a Superdex 200 Increase 10/300 GL that has a 0.01–0.6-MDa fractionation range.

### In vitro reconstitution of modular PKSs

Assays were performed in a final volume of 100 μL containing 400 mM potassium phosphate buffer, 5 mM TCEP, 9 mM MgCl_2_, 9 mM ATP, 0.75 mM 3,5-hydroxybenzoic acid, 10 mM malonate (1.6 mM malonate and 8.4 mM methylmalonate for hybrid synthase reactions), 1 mM CoA, 10 µM MatB, 8 µM VemG, and 8 µM VemH at pH 7.5. After preincubating the reaction mixture at 25 °C for 5 min, the reaction was initiated through the addition of VemG and VemH and allowed to proceed for 1–10 min. The reaction was quenched by the rapid addition of 70% v/v perchloric acid (5 µL) and then neutralized by the addition of 1 M sodium bicarbonate (25 µL). Insoluble material was removed by centrifugation at 15,000 × *g* for 5 min. The supernatant (10 µL) was subjected to HPLC analysis on a Waters 1525 HPLC system equipped with a Microsorb-MV 300–5 C_18_ column (4.6 × 250 mm). The HPLC conditions were as follows: flow rate 1 mL min^−1^; solvent A (water with 0.1% v/v formic acid) and solvent B (acetonitrile). After column equilibration with 5% B, 5–100% B over 15 min, and then 100% B for 3 min. To determine turnover numbers, six independent experiments were set up per synthase (three from one synthase prep and three from a separate prep).

### Determining turnover numbers and the apparent *K*_d_

Initial velocities (µM product/min/µM VemH) were measured with 0.2 µM VemH and varying concentrations of VemG (0.1–8.0 µM). The resulting curve was fit using GraphPad Prism 8.1 and the following quadratic Eq. 1 (since the concentration of the limiting binder, VemH, is close to the *K*_d_) to extract the turnover number and apparent *K*_d_.

1 $$y = \frac{{t\left( {c + x + K - \sqrt {\left( {c + x + K} \right)^2 - 4 \ast c \ast x} } \right)}}{{2 \ast c}}$$

where *y* = initial velocity, *c* = concentration of VemH (constant value of 0.2 µM), *x* = concentration of VemG, *K* = apparent *K*_d_, and *t* *=* turnover number.

### Scaled-up synthesis and NMR analysis of polyketides

Venemycin (**1**). IUPAC name: 6-(3,5-dihydroxyphenyl)-4-hydroxy-2*H*-pyran-2-one. Formula: C_11_H_8_O_5_. The reactor was set up with a reaction solution of 45 mL comprised of 400 mM potassium phosphate, 5 mM TCEP, 9 mM MgCl_2_, 9 mM ATP, 0.75 mM 3,5-dihydroxybenzoic acid, 10 mM malonate, and 1 mM CoA, at pH 7.5. The reaction was initiated by adding a 100-kDa MWCO dialysis bag containing Ni-NTA column purified VemG (65 µM), VemH (100 µM), and MatB (10 µM) in 2 mL. After 20 h the reaction was saturated with ammonium sulfate, extracted with EtOAc (3 × 50 mL), dried with MgSO_4_, filtered, and concentrated in vacuo. Column chromatography (SiliaFlash F60) with EtOAc/hexanes (2/1) → EtOAc as eluent. TLC (EtOAc:hexanes:HCOOH, 66:33:1): *R*_f_ = 0.35; ^1^H NMR (400 MHz, DMSO-d_6_): δ 11.77 (br.s, 1 H, 4-OH), 9.57 (s, 2 H, 9-OH, 11-OH), 6.62 (d, *J* = 2.1 Hz, 2 H, H-8, H-12), 6.47 (d, *J* = 2.0 Hz, 1 H, H-5), 6.30 (t, *J* = 2.2 Hz, 1 H, H-10), 5.31 (m, 1 H, H-3) (D_2_O suppressed). ^1^H NMR (400 MHz, D_2_O): δ 10.06 (s, 2 H, 9-OH, 11-OH), 6.76 (d, *J* = 2.2 Hz, 2 H, H-8, H-12), 6.36 (t, *J* = 2.2 Hz, 1 H, H-10), 6.30 (d, *J* = 2.0 Hz, 1 H, H-5), 5.01 (d, *J* = 2.0 Hz, 1 H, H-3). ^13^C NMR (400 MHz, DMSO-d_6_): δ 171.2 (C2), 163.6 (C4), 160.6 (C6), 159.3 (C9, C11), 133.2 (C7), 105.6 (C10), 103.9 (C8, C12), 98.6 (C5), 89.9 (C3).

Pyrone (**2**). IUPAC name: 6-ethyl-4-hydroxy-2*H*-pyran-2-one. Formula: C_7_H_8_O_3_. The reactor was set up with a reaction solution of 45 mL comprised of 400 mM potassium phosphate, 5 mM TCEP, 12 mM MgCl_2_, 12 mM ATP, 1.6 mM malonate, and 8.4 mM methylmalonate at pH 7.5. The reaction was initiated by adding a 100 kDa MWCO dialysis bag containing Ni-NTA column purified PikAI/VemG (60 µM), VemH (100 µM), and MatB (10 μM) in 2 mL. After 2 d the reaction was saturated with ammonium sulfate, extracted with EtOAc (3 × 50 mL), dried with MgSO_4_, filtered, and concentrated in vacuo. Column chromatography (SiliaFlash F60) with EtOAc/hexanes (1/1) → (2/1) with 1% formic acid as eluent. TLC (EtOAc:hexanes:HCOOH, 66:33:1): *R*_f_ = 0.53; ^1^H NMR (500 MHz, CDCl_3_): δ 5.96 (m, 1 H, H-5), 5.56 (d, *J* = 2.2 Hz, 1 H, H-3), 2.53 (q, *J* = 7.5 Hz, 2 H, H_2_-7), 1.22 (t, *J* = 7.5 Hz, 3 H, H_3_-8). ^1^H NMR (400 MHz, D_2_O): δ 5.96 (d, *J* = 1.0 Hz, 1 H, H-5), 5.20 (s, 1 H, H-3), 2.50 (q, *J* = 7.5 Hz, 2 H, H_2_-7), 1.17 (t, 3 H, H_3_-8). ^13^C NMR (CDCl_3_): δ 172.3 (C2), 168.5 (C4), 167.8 (C6), 100.3 (C5), 89.8 (C3), 26.9 (C7), 10.8 (C8).

Methylvenemycin (**3**). IUPAC name: 6-(3,5-dihydroxyphenyl)-4-hydroxy-3-methyl-2*H*-pyran-2-one. Formula: C_12_H_10_O_5_. The reactor was set up with a reaction solution of 45 mL comprised of 400 mM potassium phosphate, 5 mM TCEP, 9 mM MgCl_2_, 9 mM ATP, 0.75 mM 3,5-dihydroxybenzoic acid, 1.6 mM malonate and 8.4 mM methylmalonate at pH 7.5. The reaction was initiated by adding a 100 kDa MWCO dialysis bag containing Ni-NTA column purified VemG (71 µM), VemH/PikAIV (100 µM), and MatB (10 µM) in 2 mL. After 2 d the reaction was saturated with ammonium sulfate, extracted with EtOAc (3 × 50 mL), dried with MgSO_4_, filtered, and concentrated in vacuo. Column chromatography (SiliaFlash F60) with EtOAc/hexanes (1/1) → (2/1) with 1% formic acid as eluent. TLC (EtOAc:hexanes:HCOOH, 66:33:1) *R*_f_ = 0.45; ^1^H NMR (400 MHz, DMSO-d_6_): δ 9.55 (s, 2 H, 9-OH, 11-OH), 6.55 (d, *J* = 2.2 Hz, 2 H, H-8, H-12), 6.44 (s, 1 H, H-5), 6.26 (t, *J* = 2.1 Hz, 1 H, H-10), 1.76 (s, 3 H, CH_3_). ^1^H NMR (400 MHz, D_2_O): δ 10.05 (s, 2 H, 9-OH, 11-OH), 6.76 (d, *J* = 2.2 Hz, 2 H, H-8, H-12), 6.35 (m, 2 H, H-5, H-10), 1.67 (s, 3 H, CH_3_). ^13^C NMR (400 MHz, DMSO-d_6_): δ 164.8 (C2), 159.4 (C4, C9, C11), 157.0 (C6), 133.5 (C7), 105.2 (C10), 103.5 (C8, C12), 98.5 (C5), 98.3 (C3), 9.2 (CH_3_).

Methylpyrone (**4**). IUPAC name: 6-ethyl-4-hydroxy-3-methyl-2*H*-pyran-2-one. Formula: C_8_H_10_O_3_. The reactor was set up with a reaction solution of 45 mL comprised of 400 mM potassium phosphate, 5 mM TCEP, 12 mM MgCl_2_, 12 mM ATP, 1.6 mM malonate, and 8.4 mM methylmalonate at pH 7.5. The reaction was initiated by adding a 100-kDa MWCO dialysis bag containing Ni-NTA column purified PikAI/VemG (124 µM), VemH/PikAIV (208 µM), and MatB (10 µM) in 2 mL. After 5 d the reaction was saturated with ammonium sulfate, extracted with EtOAc (3 × 50 mL), dried with MgSO_4_, filtered, and concentrated in vacuo. Less than one milligram of **4** was obtained. Column chromatography (SiliaFlash F60) with EtOAc/hexanes (1/1) → (2/1) with 1% formic acid as eluent. ^1^H NMR (500 MHz, CDCl_3_): δ 5.71 (s, 1 H, H-5), 2.50 (q, *J* = 7.5 Hz, 2 H, H_2_-7), 1.91 (s, 3 H, CH_3_), 1.25 (t, *J* = 7.5 Hz, 3 H, H_3_-8).

Deshydroxyvenemycin (**5**). IUPAC name: 4-hydroxy-6-(3-hydroxyphenyl)-2*H*-pyran-2-one. Formula: C_11_H_8_O_4_. The reactor was set up with a reaction solution of 45 mL comprised of 400 mM potassium phosphate, 5 mM TCEP, 9 mM MgCl_2_, 9 mM ATP, 0.75 mM 3-hydroxybenzoic acid, and 10 mM malonate at pH 7.5. The reaction was initiated by adding a 100 kDa MWCO dialysis bag containing Ni-NTA column purified VemG (65 µM), VemH (100 µM), and MatB (10 µM) in 2 mL. After 1 d the reaction was saturated with ammonium sulfate, extracted with EtOAc (3 × 50 mL), dried with MgSO_4_, filtered, and concentrated in vacuo. Column chromatography (SiliaFlash F60) with EtOAc/hexanes (1/1) → (2/1) as eluent. TLC (EtOAc:hexanes:HCOOH, 66:33:1): *R*_f_ = 0.50; ^1^H NMR (400 MHz, DMSO-d_6_): δ 9.76 (s, 1 H, OH), 7.29 (t, *J* = 7.4 Hz, 1 H, H-11), 7.26 (d, *J* = 7.4 Hz, 1 H, H-12), 7.18 (t, *J* = 2.1 Hz, 1 H, H-8), 6.88 (d, *J* = 7.4 Hz, 1 H, H-10), 6.55 (s, 1 H, H-5), 5.22 (s, 1 H, H-3). ^1^H NMR (400 MHz, D_2_O/DMSO-*d*_6_ 90/10): δ 7.32–7.26 (m, 2 H, H-11, H-12), 7.19 (s, 1 H, H-8), 6.89 (dt, *J* = 6.3, 2.6 Hz, 1 H, H-10), 6.51 (s, 1 H, H-5), 4.99 (s, 1 H, H-3). ^13^C-NMR (400 MHz, DMSO-d_6_): δ 171.8 (C2), 164.2 (C4), 160.4 (C6), 158.1 (C9), 132.8 (C7), 130.8 (C11), 118.5 (C10), 116.8 (C12), 112.4 (C8), 99.3 (C5), 89.8 (C3).

Deshydroxymethylvenemycin (**6**). IUPAC name: 4-hydroxy-6-(3-hydroxyphenyl)-3-methyl-2*H*-pyran-2-one. Formula: C_12_H_10_O_4_. The reactor was set up with a reaction solution of 45 mL comprised of 400 mM potassium phosphate, 5 mM TCEP, 9 mM MgCl_2_, 9 mM ATP, 0.75 mM 3-hydroxybenzoic acid, 1.4 mM malonate, and 8.4 mM methylmalonate at pH 7.5. The reaction was initiated by adding a 100-kDa MWCO dialysis bag containing Ni-NTA column purified VemG (69 µM), VemH/PikAIV (100 µM), and MatB (10 µM) in 2 mL. After 2 d the reaction was saturated with ammonium sulfate, extracted with EtOAc (3 × 50 mL), dried with MgSO_4_, filtered, and concentrated in vacuo. Column chromatography (SiliaFlash F60) with EtOAc/hexanes (1/2) → (1/1) as eluent. TLC (EtOAc:hexanes:HCOOH, 66:33:1 v/v): *R*_f_ = 0.60; ^1^H NMR (500 MHz, DMSO-d_6_): δ 9.80 (s, 1 H, 9-OH), 7.31 (t, *J* = 8.0 Hz, 1 H, H-11), 7.18 (d, *J* = 7.7 Hz, 1 H, H-12), 7.14 (s, 1 H, H-8), 6.88 (d, 1 H, *J* = 8.2 Hz, H-10), 6.63 (s, 1 H, H-5), 1.83 (s, 3 H, CH_3_). ^1^H NMR (400 MHz, D_2_O/DMSO-d_6_ 90/10): δ 7.26–7.22 (m, 2 H, H-11, H-12), 7.16 (s, 1 H, H-8), 6.86–6.80 (m, 1 H, H-10), 6.41 (s, 1 H, H-5), 1.68 (s, 3 H, CH_3_). ^13^C-NMR (500 MHz, DMSO-d_6_): δ 165.5 (C2), 164.7 (C4), 158.3 (C6), 157.0 (C9), 132.9 (C7), 130.7 (C11), 118.1 (C10), 116.2 (C12), 112.0 (C8), 98.8 (C3), 98.3 (C5), 9.1 (CH_3_).

### Constructing the venemycin synthase

Several solved structures were combined to generate the homodimeric synthase comprised of the 232-kDa VemG and the 140-kDa VemG (Supplementary Fig. 1). The structures of each of the domains were generated using the Fold and Function Assignment System (FFAS) server^29^. The A domains were threaded through a dimeric long-chain fatty acid CoA ligase (PDB 3G7S). KR^0^ was threaded through the monomeric ketoreductase from the second module of the erythromycin assembly line (PDB 2FR0). KS and AT domains were threaded through the dimeric KS and monomeric AT domains from the third and fourth modules of the erythromycin assembly line (PDB 6C9U). ACP domains were threaded through the monomeric ACP domain from the eighth module of the mycolactone assembly line (in MlsB, PDB 6H0Q). The TE domain was threaded through the dimeric TE domain from the tautomycetin assembly line (PDB 3LCR). The docking domains were threaded through the dimeric docking domains connecting the second and third polypeptides of the erythromycin assembly line (PDBs 1PZR and 1PZQ). Domains were relatively oriented using the program Pymol (Schrödinger, LLC), and flexible loop residues were built with the program Coot^30^.

### Reporting summary

Further information on research design is available in the Nature Research Reporting Summary linked to this article.

## Supplementary information

Supplementary Information

Reporting Summary

Description of Additional Supplementary Files

Supplementary Data 1

## Data Availability

Data supporting the findings of this work are available within the paper and its Supplementary Information files. A reporting summary for this Article is available as a Supplementary Information file. Data generated and analyzed herein are available from the corresponding author upon request. The coordinates for the atomic model of the venemycin synthase can be downloaded from http://keatinge-clay.cm.utexas.edu/research/VemGH_model.pdb. The X-ray crystallographic coordinates for structures reported in this study have been deposited at the Cambridge Crystallographic Data Centre (CCDC), under deposition numbers 1921205 (**1**) [10.5517/ccdc.csd.cc22h5c4], 1921202 (**2**) [10.5517/ccdc.csd.cc22h581], 1921204 (**3**) [10.5517/ccdc.csd.cc22h5b3], 1921206 (**5**) [10.5517/ccdc.csd.cc22h5d5], and 1921203 (**6**) [10.5517/ccdc.csd.cc22h592]. These data can be obtained free of charge from CCDC via www.ccdc.cam.ac.uk/data_request/cif. The source data underlying Figs. 2 and 4 are provided as a Source Data file.

## Source data

Source Data
