# Supplementary material for: An in vitro platform for engineering and harnessing modular polyketide synthases
Source: Nat Commun. 2020 Jan 3;11:80. doi: 10.1038/s41467-019-13811-0 (PMC6941969; doi:10.1038/s41467-019-13811-0)
Supplement: Supplementary file 1 — Supplementary Information [file 41467_2019_13811_MOESM1_ESM.pdf]

An *in vitro* platform for engineering and harnessing modular polyketide synthases

Miyazawa *et al.*

Supplementary Methods

Crystallographic structure determination of polyketide products

X-ray crystal structure analysis of venemycin (**1**)**.** Crystals grew as clusters of long, colorless needles by slow evaporation from 90% methanol and 10% water. The data crystal was cut from a larger crystal and had the approximate dimensions of 0.14 x 0.015 x 0.015 mm. The data were collected on an Agilent Technologies SuperNova Dual Source diffractometer using a μ-focus Cu K_α radiation source (λ = 1.5418 Å) with collimating mirror monochromators. A total of 508 frames of data were collected using ω-scans with a scan range of 1° and a counting time of 27.5 s per frame with a detector offset of +/- 42.7° and 85 s per frame with a detector offset of 110.8°. The data were collected at 100 K using an Oxford 700 Cryostream low temperature device. Data collection, unit cell refinement, and data reduction were performed using Rigaku Oxford Diffraction's CrysAlisPro V 1.171.40.37a. The structure was solved by direct methods using SHELXT[1] and refined by full-matrix least-squares on F^2 with anisotropic displacement parameters for the non-H atoms using SHELXL-2016/6[2]. Structure analysis was aided by use of the programs PLATON[3], OLEX2[4], and WinGX[5]. The hydrogen atoms were calculated in ideal positions with isotropic displacement parameters set to 1.2xUeq of the attached atom (1.5xUeq for methyl hydrogen atoms). The hydrogen atoms on the water molecules were initially assigned by a best fit to the electron density around the oxygen atoms. These hydrogen atoms were subsequently fixed with O-H bond lengths set to 0.84 Å. However, one of the water molecules, O7, was within H-bonding distance to a symmetry related O7, where one of the H atoms on each was directed towards each other. This required that this H atom, H7B, is disordered. In subsequent refinement models, the site occupancy factor for H7B was fixed at ½ occupancy. Equation 1 was minimized (Equations **1**-**3**). $R_w(F^2)$ refined to 0.2352, with an R(F) of 0.08398 and a goodness of fit, S, of 1.02. Definitions used for calculating R(F), $R_w(F^2)$, and the goodness of fit, S, are provided (Equations **4**-**6**). The data were checked for secondary extinction effects but no correction was necessary. Neutral atom scattering factors and values used to calculate the linear absorption coefficient are from the International Tables for X-ray Crystallography[6].

X-ray crystal structure analysis of pyrone (**2**)**.** Crystals grew as clusters of colorless plates by slow evaporation from 80% EtOAc, 10% methanol, and 10% water. The data crystal was separated from a cluster of crystals and had the approximate dimensions of 0.20 x 0.11 x 0.04 mm. The data were collected on an Agilent Technologies SuperNova Dual Source diffractometer using a μ-focus Cu K_α radiation source (λ = 1.5418 Å) with collimating mirror monochromators. A total of 1163 frames of data were collected using ω-scans with a scan range of 1° and a counting time of 14.5 s per frame for frames

collected with a detector offset of +/- 42.4° and 47 s per frame with frames collected with a detector offset of 110.8°. The data were collected at 100 K using an Oxford Cryostream low temperature device. Data collection, unit cell refinement, and data reduction were performed using Rigaku Oxford Diffraction's CrysAlisPro V 1.171.40.37a. The structure was solved by direct methods using SHELXT[1] and refined by full-matrix least-squares on F^2 with anisotropic displacement parameters for the non-H atoms using SHELXL-2016/6[2]. Structure analysis was aided by use of the programs PLATON[3], OLEX2[4], and WinGX[5]. The hydrogen atoms on the carbon atoms were calculated in ideal positions with isotropic displacement parameters set to 1.2xUeq of the attached atom (1.5xUeq for methyl hydrogen atoms). Equation 1 was minimized (Equations **1**, **3,** and **7**). $R_w(F^2)$ refined to 0.240, with an R(F) of 0.0776 and a goodness of fit, S, of 1.06. Definitions used for calculating R(F), $R_w(F^2)$, and the goodness of fit, S, are provided (Equations **4**-**6**). The data were checked for secondary extinction effects but no correction was necessary. Neutral atom scattering factors and values used to calculate the linear absorption coefficient are from the International Tables for X-ray Crystallography[6].

X-ray crystal structure analysis of methylvenemycin (**3**)**.** Crystals grew as clusters of thin colorless needles by slow evaporation from 90% methanol and 10% water. The data crystal was separated from a cluster of crystals and had the approximate dimensions of 0.27 x 0.043 x 0.022 mm. The data were collected on an Agilent Technologies SuperNova Dual Source diffractometer using a μ-focus Cu K_α radiation source (λ = 1.5418 Å) with collimating mirror monochromators. A total of 482 frames of data were collected using ω-scans with a scan range of 1° and a counting time of 54 s per frame for frames collected with a detector offset of +/- 41.6° and 142.5 s per frame with frames collected with a detector offset of 110.7°. The data were collected at 100 K using an Oxford Cryostream low temperature device. Data collection, unit cell refinement, and data reduction were performed using Rigaku Oxford Diffraction's CrysAlisPro V 1.171.40.37a. The structure was solved by direct methods using SHELXT[1] and refined by full-matrix least-squares on F^2 with anisotropic displacement parameters for the non-H atoms using SHELXL-2016/6[2]. Structure analysis was aided by use of the programs PLATON[3], OLEX2[4], and WinGX[5]. The hydrogen atoms were calculated in ideal positions with isotropic displacement parameters set to 1.2xUeq of the attached atom (1.5xUeq for methyl hydrogen atoms). The data crystal was twinned. The twin law was determined using PLATON. The twin fraction refined to 0.482(4). Equation **1** was minimized (Equations **1**, **3**, and **8**). $R_w(F^2)$ refined to 0.331, with an R(F) of 0.126 and a goodness of fit, S, of 1.17. Definitions used for calculating R(F), $R_w(F^2)$ and the goodness of fit, S, are provided (Equations **4**-**6**). The data were checked for secondary extinction effects but no correction was necessary. Neutral atom scattering factors and values used to calculate the linear absorption coefficient are from the International Tables for X-ray Crystallography[6].

X-ray crystal structure analysis of deshydroxyvenemycin (**5**). Crystals grew as clusters of very small, colorless needles by slow evaporation from 90% methanol and 10% water. The data crystal was broken from a cluster and had the approximate dimensions of 0.12 x 0.058 x 0.036 mm. The data were collected on an Agilent Technologies SuperNova Dual Source diffractometer using a μ-focus Cu K_{α} radiation source (λ = 1.5418 Å) with collimating mirror monochromators. A total of 704 frames of data were collected using ω-scans with a scan range of 1° and a counting time of 41 s per frame with a detector offset of +/- 42.4° and 151 s per frame with a detector offset of 110.2°. The data were collected at 100 K using an Oxford 700 Cryostream low temperature device. Data collection, unit cell refinement, and data reduction were performed using Rigaku Oxford Diffraction's CrysAlisPro V 1.171.40.37a. The structure was solved by direct methods using SHELXT[1] and refined by full-matrix least-squares on F^2 with anisotropic displacement parameters for the non-H atoms using SHELXL-2016/6[2]. Structure analysis was aided by use of the programs PLATON[3], OLEX2[4], and WinGX[5]. The hydrogen atoms were calculated in ideal positions with isotropic displacement parameters set to 1.2xUeq of the attached atom (1.5xUeq for methyl hydrogen atoms). The phenol ring was disordered by an approximately 180º rotation about the C-C bond connecting the two ring systems. The disorder was modeled using OLEX2. The site occupancy for the major component refined to 79(1)%. Equation **1** was minimized (Equations **1**, **3,** and **9**). $R_w(F^2)$ refined to 0.278, with an R(F) of 0.0923 and a goodness of fit, S, of 1.05. Definitions used for calculating R(F), $R_w(F^2)$ and the goodness of fit, S, are provided (Equations **4**-**6**). The data were checked for secondary extinction effects but no correction was necessary. Neutral atom scattering factors and values used to calculate the linear absorption coefficient are from the International Tables for X-ray Crystallography[6].

X-ray crystal structure analysis of deshydroxymethylvenemycin (**6**). Crystals grew as clusters of thin, colorless prisms by slow evaporation from 90% methanol and 10% water. The data crystal had the approximate dimensions of 0.21 x 0.15 x 0.038 mm. The data were collected on an Agilent Technologies SuperNova Dual Source diffractometer using a μ-focus Cu K_{α} radiation source (λ = 1.5418 Å) with collimating mirror monochromators. A total of 388 frames of data were collected using ω-scans with a scan range of 1° and a counting time of 30.5 s per frame with a detector offset of 0.0° and 91.5 s per frame with a detector offset of 82.6°. The data were collected at 100 K using an Oxford 700 Cryostream low temperature device. Data collection, unit cell refinement and data reduction were performed using Rigaku Oxford Diffraction's CrysAlisPro V 1.171.40.37a. The structure was solved by direct methods using SHELXT[1] and refined by full-matrix least-squares on F^2 with anisotropic displacement parameters for the non-H atoms using SHELXL-2016/6[2]. Structure analysis was aided by use of the programs PLATON[3], OLEX2[4], and WinGX[5]. The hydrogen atoms were calculated in ideal positions with

isotropic displacement parameters set to 1.2xUeq of the attached atom (1.5xUeq for methyl hydrogen atoms). Equation **1** was minimized (Equations **1**, **3**, and **10**). $R_w(F_2)$ refined to 0.2472, with an R(F) of 0.0850, and a goodness of fit, S, of 1.04. Definitions used for calculating R(F), $R_w(F^2)$ and the goodness of fit, S, are provided (Equations **4-6**). The data were checked for secondary extinction effects but no correction was necessary. Neutral atom scattering factors and values used to calculate the linear absorption coefficient are from the International Tables for X-ray Crystallography[6].

Equations:

$\Sigma w(|F_O|^2 - |F_C|^2)^2$ (**1**)

$w = 1/[(\sigma(F_o))^2 + (0.0717*P)^2]$ (**2**)

$P = (|F_o|^2 + 2|F_c|^2)/3$ (**3**)

$R_W(F^2) = \{\Sigma w(|F_o|^2 - |F_c|^2)^2/\Sigma w(|F_o|)^4\}^{1/2}$ (**4**)
where w is weight given each reflection

$R(F) = \Sigma(|F_o| - |F_c|)/\Sigma|F_o|\}$ for reflections with $F_o > 4(\sigma(F_o))$ (**5**)

$S = [\Sigma w(|F_o|^2 - |F_c|^2)^2/(n - p)]^{1/2}$ (**6**)
where n is the number of reflections and p is the number of refined parameters

$w = 1/[(\sigma(F_o))^2 + (0.1135*P)^2 + (0.10739*P)]$ (**7**)

$w = 1/[(\sigma(F_o)) + (0.12*P)^2]$ (**8**)

$w = 1/[(\sigma(F_o))^2 + (0.1208*P)^2]$ (**9**)

$w = 1/[(\sigma(F_o))^2 + (0.1318*P)^2 + (1.5455*P)]$ (**10**)

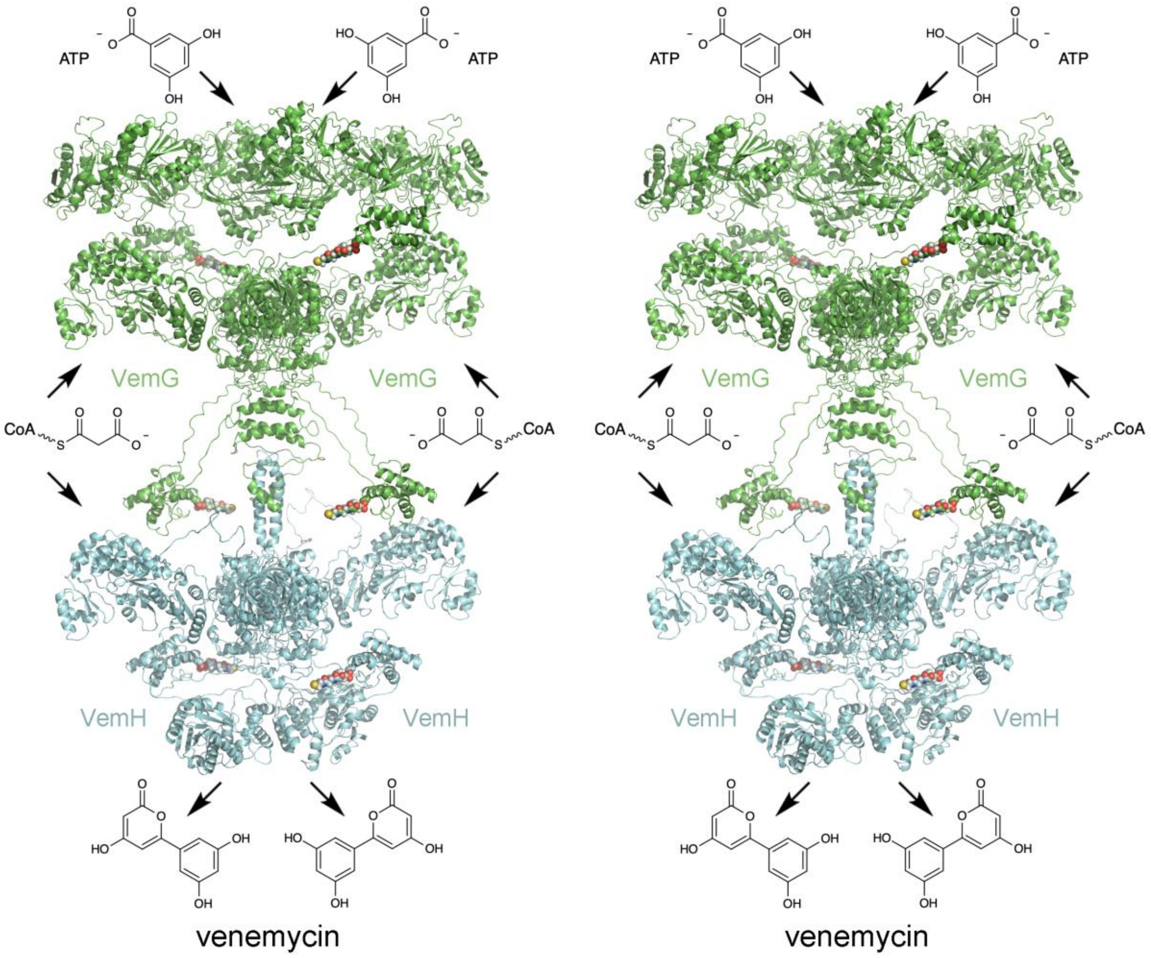

Supplementary Figure 1. All-atom model of the venemycin assembly line. A wall-eyed stereodiagram shows a model of VemG (green) and VemH (cyan) within the venemycin assembly line. All residues, including those of flexible linkers, are included. The phosphopantetheinyl arms of ACP domains are represented in spheres. Coordinates can be downloaded at http://keatinge-clay.cm.utexas.edu/research/VemGH_model.pdb.

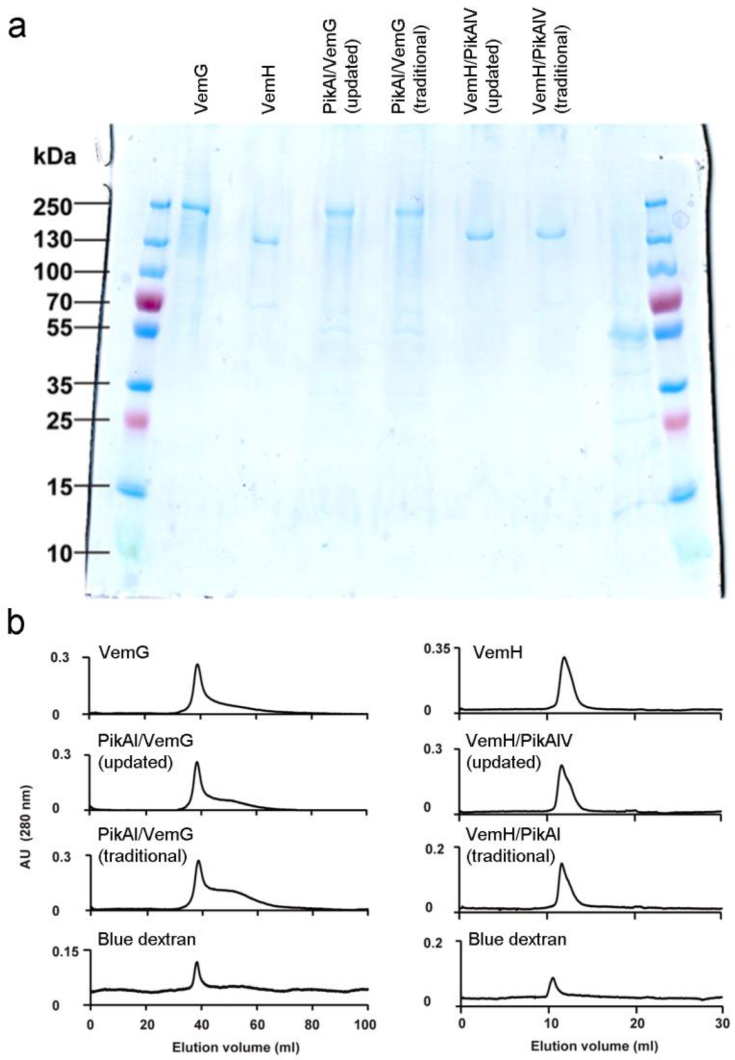

Supplementary Figure 2. Analysis of protein quality. a) SDS-PAGE gel of VemG, VemH, hybrids, and MatB. Lane 1 - VemG, 232 kDa; lane 2 - VemH, 140 kDa; lane 3 - PikAI/VemG (updated), 233 kDa; lane 4 - PikAI/VemG (traditional), 233 kDa; lane 5 - VemH/PikAIV (updated), 144 kDa; lane 6 - VemH/PikAIV (traditional), 143 kDa; lane 7, MatB (53 kDa). PageRuler Plus Prestained Protein Ladder, 4-20% Tris-glycine gel (Thermo Fisher Scientific). b) Size exclusion chromatography. VemG and PikAIV/VemG hybrids were analyzed by Sephacryl S-300 HR column equilibrated with size exclusion buffer (50 mM potassium phosphate, 150 mM NaCl, 1 mM TCEP, 10% v/v glycerol, pH 7.5. VemH and VemH/PikAIV hybrids were analyzed by Superdex 200 Increase 16/600 GL column equilibrated with size exclusion buffer. Blue dextran indicates the void volumes for both columns.

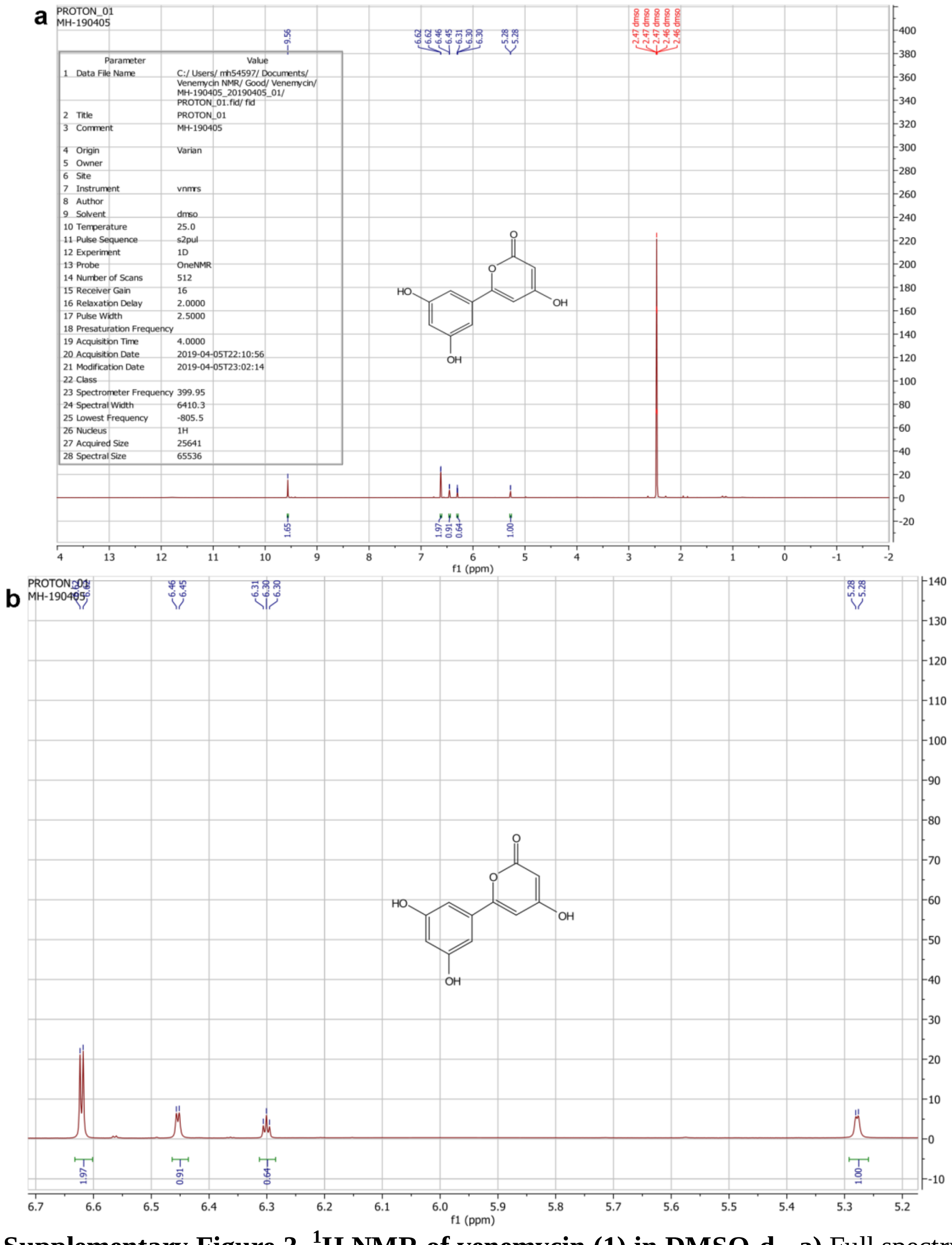

Supplementary Figure 3. ^{1}H NMR of venemycin (1) in DMSO-d$_6$. a) Full spectrum. **b)** Expanded region.

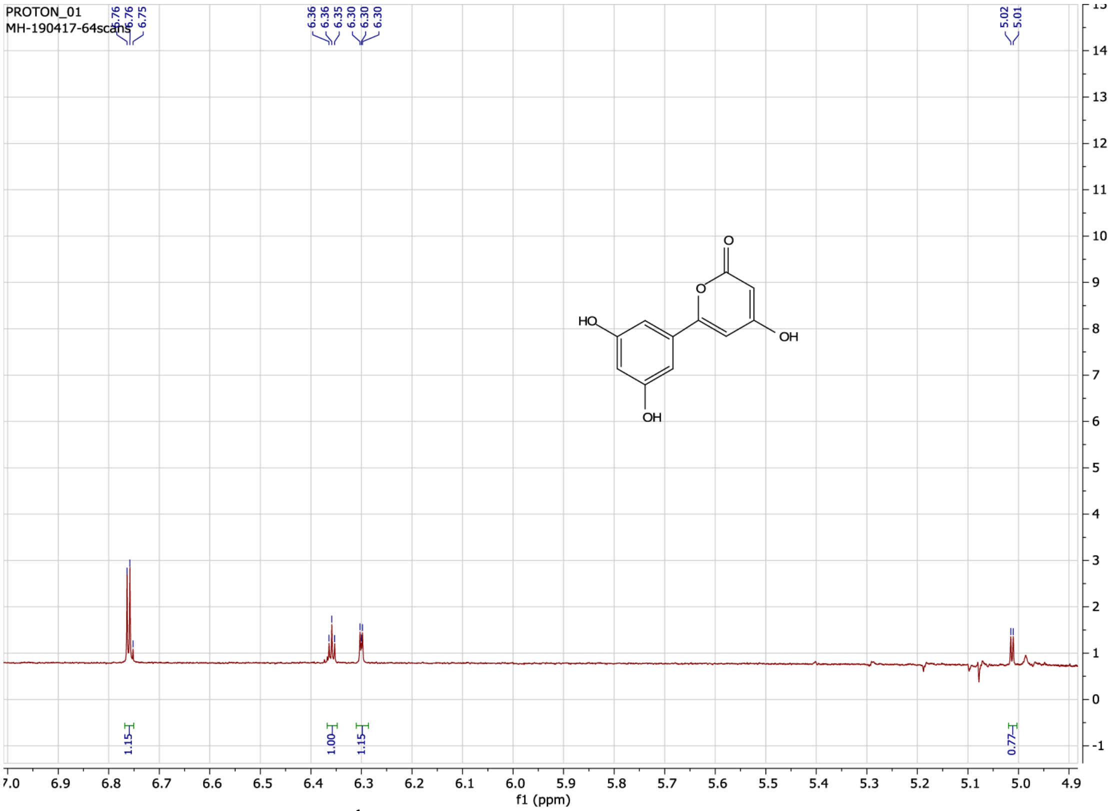

Supplementary Figure 4. ^{1}H NMR of venemycin (1) in D_2O.

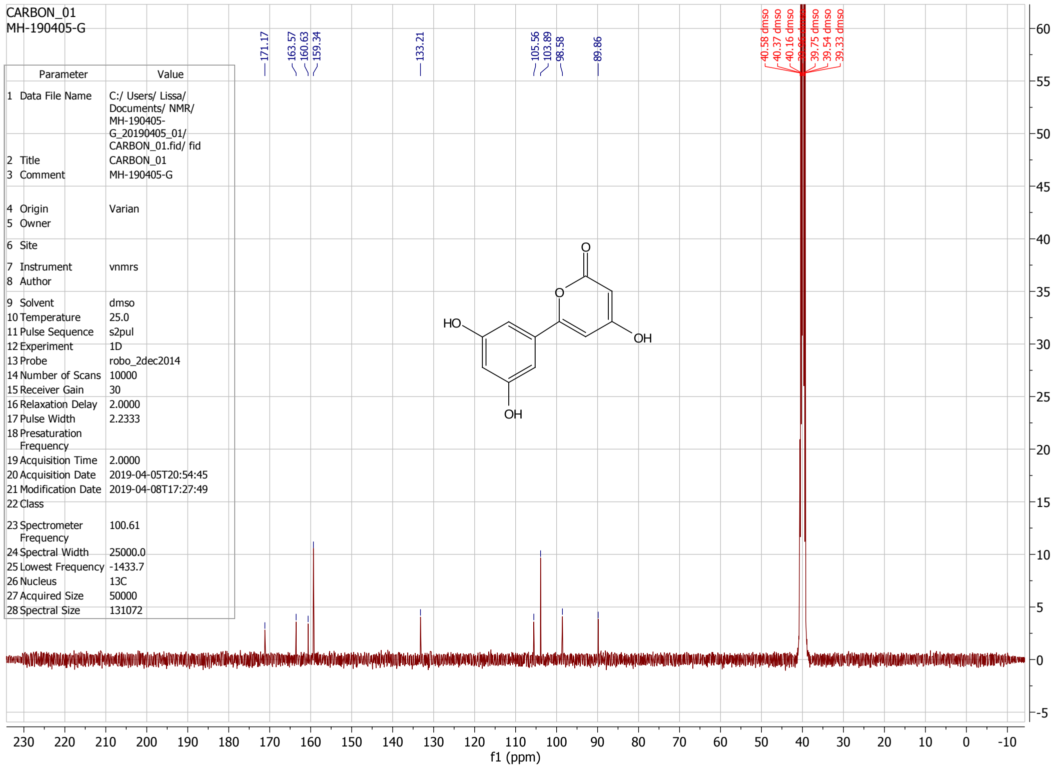

Supplementary Figure 5. ^{13}**C NMR of venemycin (1) in DMSO-d$_6$.**

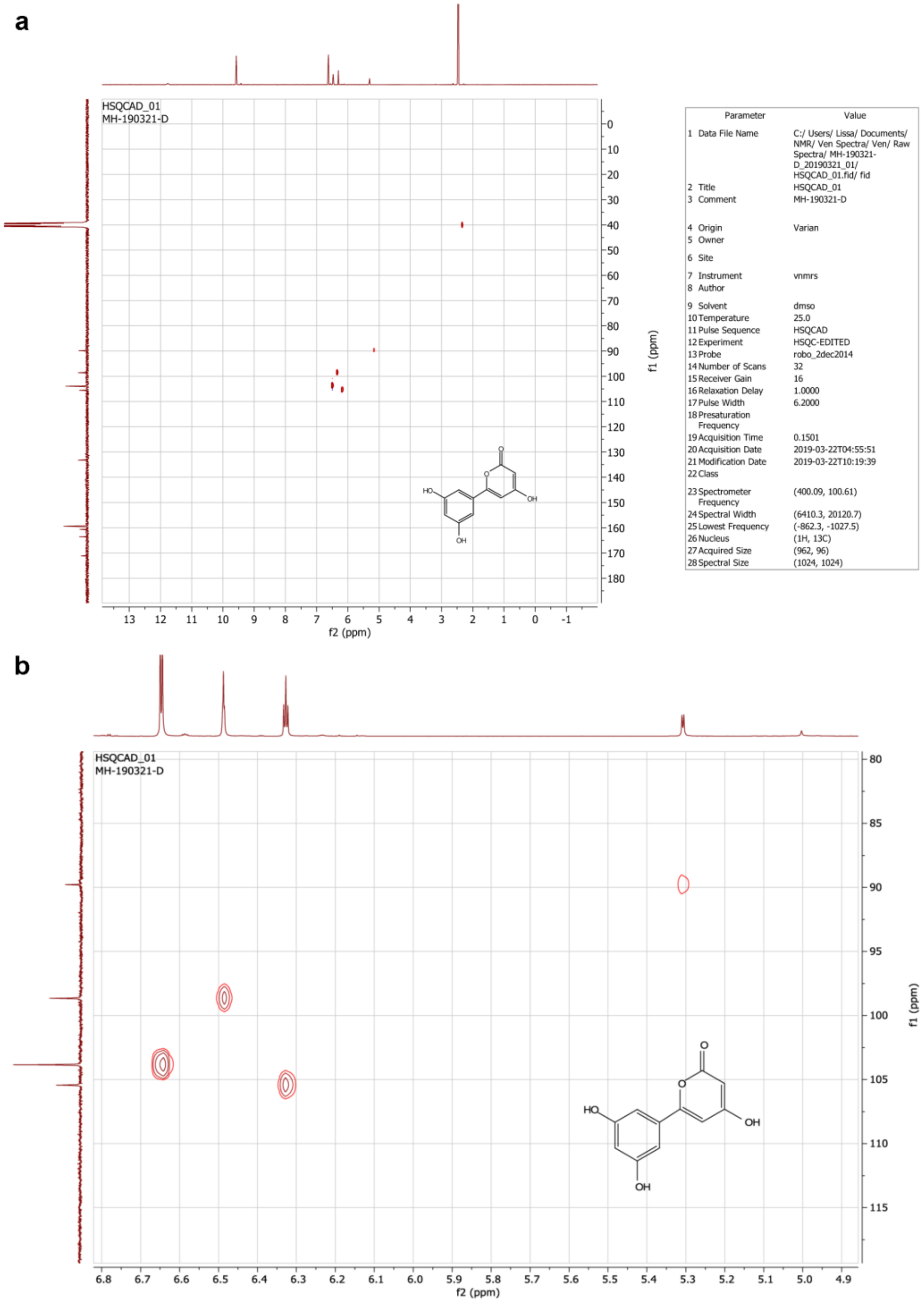

Supplementary Figure 6. ^{1}H-^{13}C-HSQC of venemycin (1) in DMSO-d_6. a) Full spectrum. **b)** Expanded region.

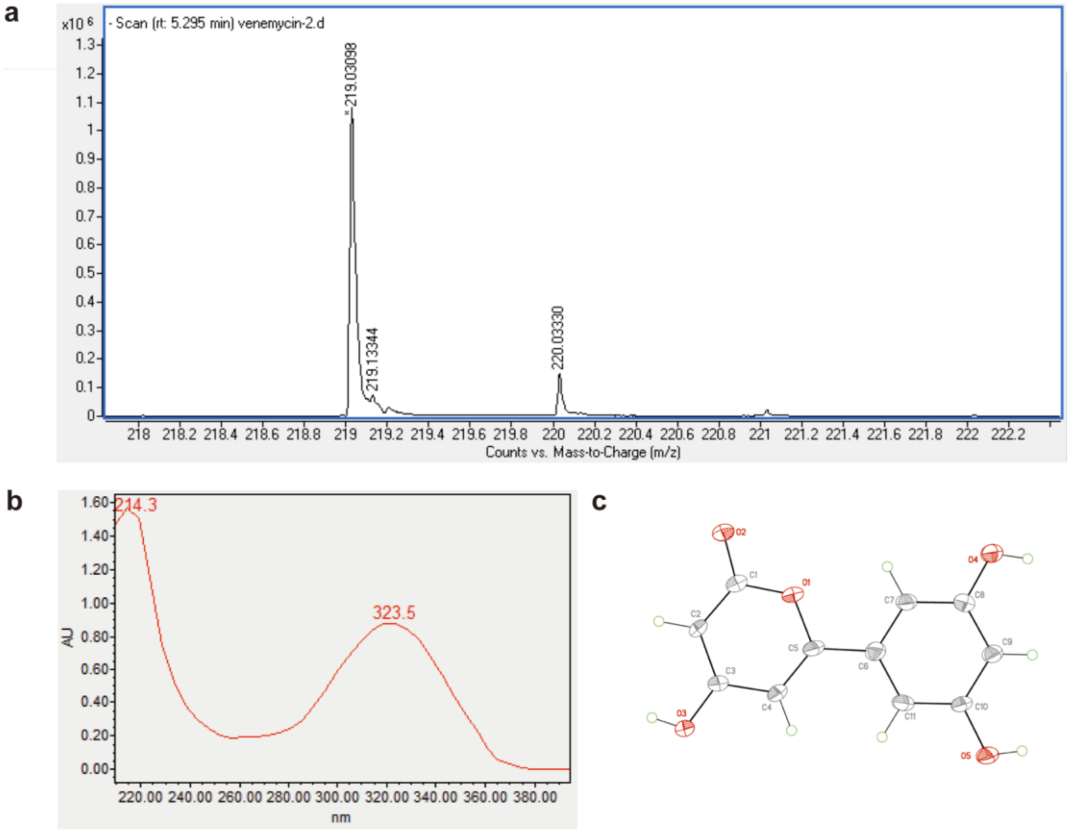

Supplementary Figure 7. Additional characterization of venemycin (1). a) High resolution MS. [M-H]$^{-}$ 219.0299 *m*/*z* calculated, 219.0310 *m*/*z* found . **b)** Absorbance spectrum. **c)** X-ray crystal structure.

a

b

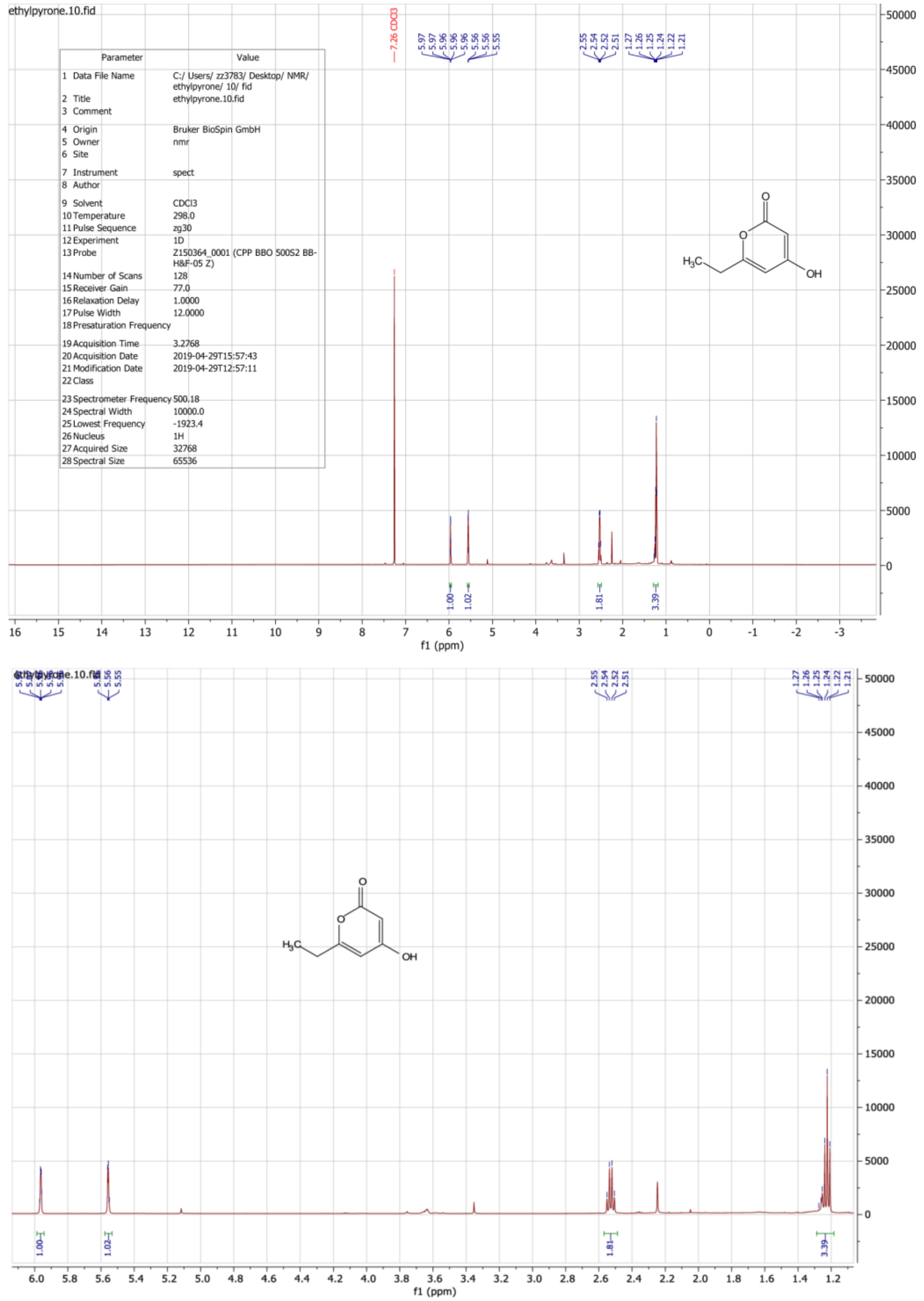

Supplementary Figure 8. ^{1}H NMR of pyrone (2) in $CDCl_3$. a) Full spectrum. **b)** Expanded region.

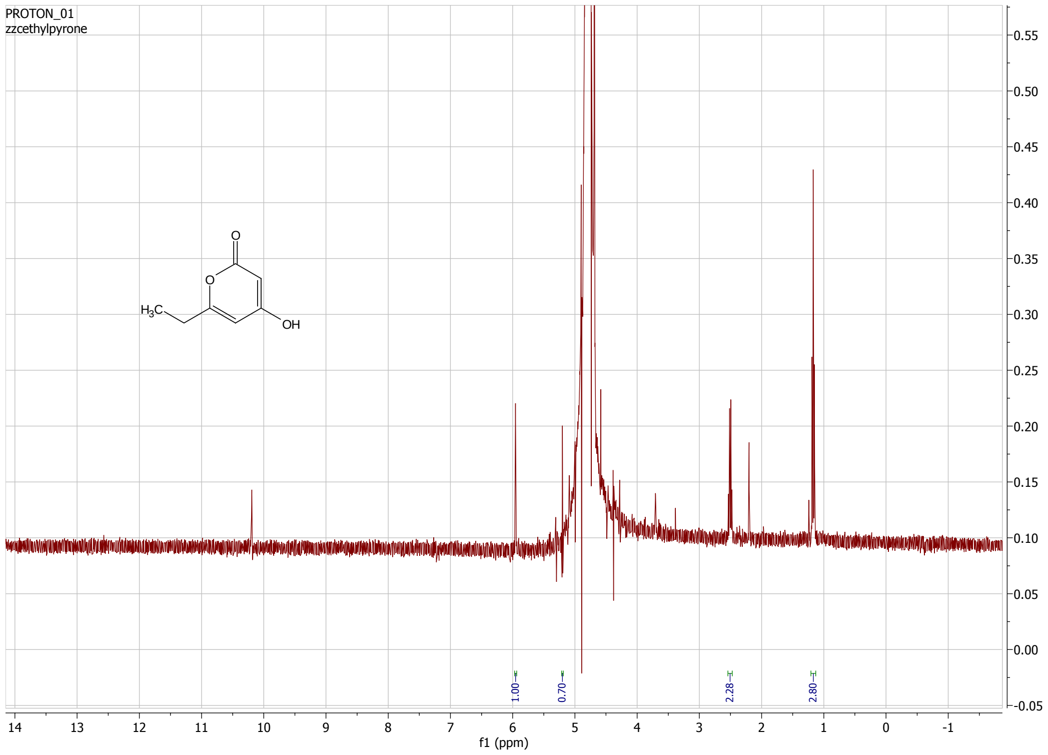

Supplementary Figure 9. ^{1}H NMR of pyrone (2) in D_2O.

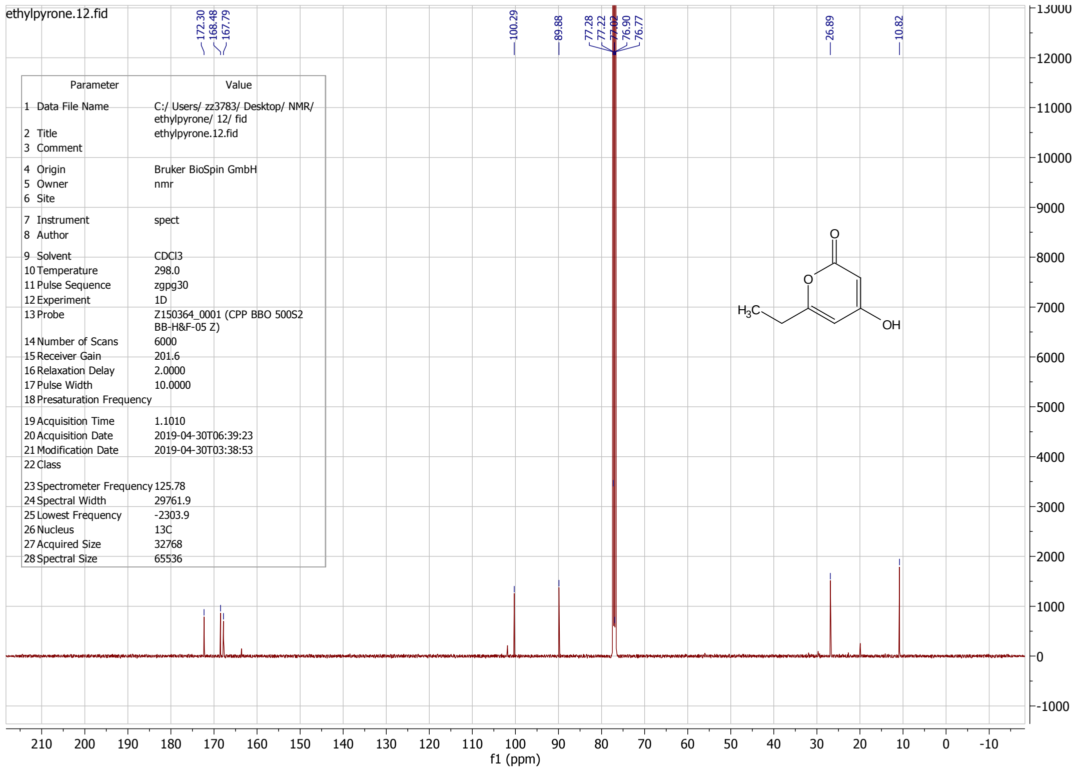

Supplementary Figure 10. ^{13}C NMR of pyrone (2) in $CDCl_3$.

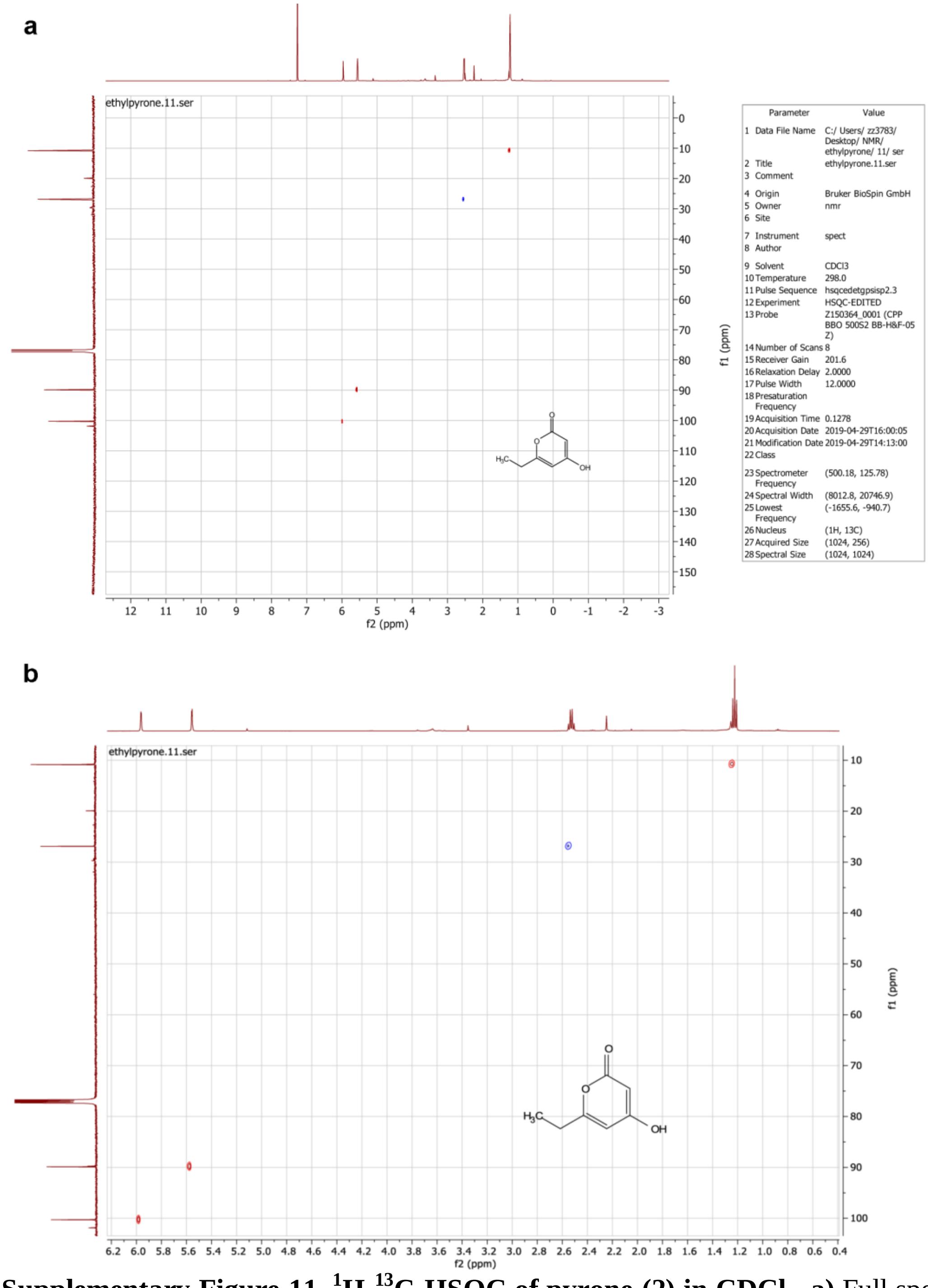

Supplementary Figure 11. ^{1}H-^{13}C-HSQC of pyrone (2) in $CDCl_3$. a) Full spectrum. **b)** Expanded region.

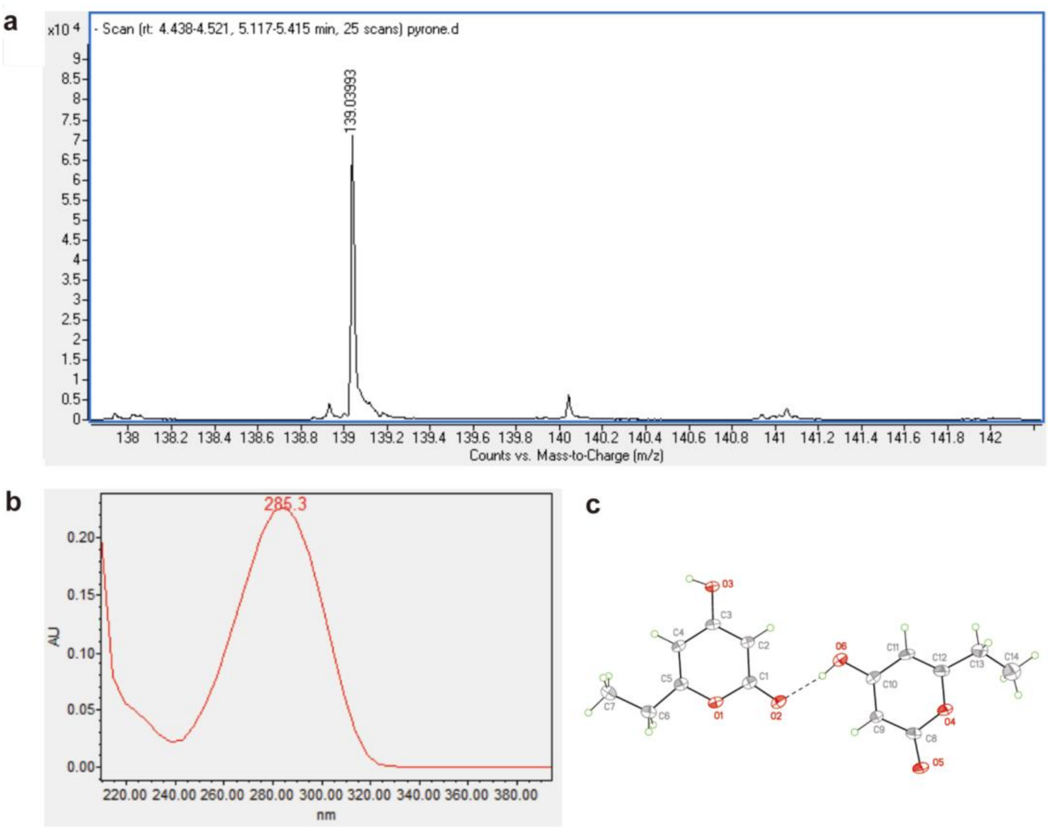

Supplementary Figure 12. Additional characterization of pyrone (2). a) High resolution MS. $[M-H]^-$ 139.0401 *m/z* calculated, 139.0399 *m/z* found. **b)** Absorbance spectrum. **c)** X-ray crystal structure.

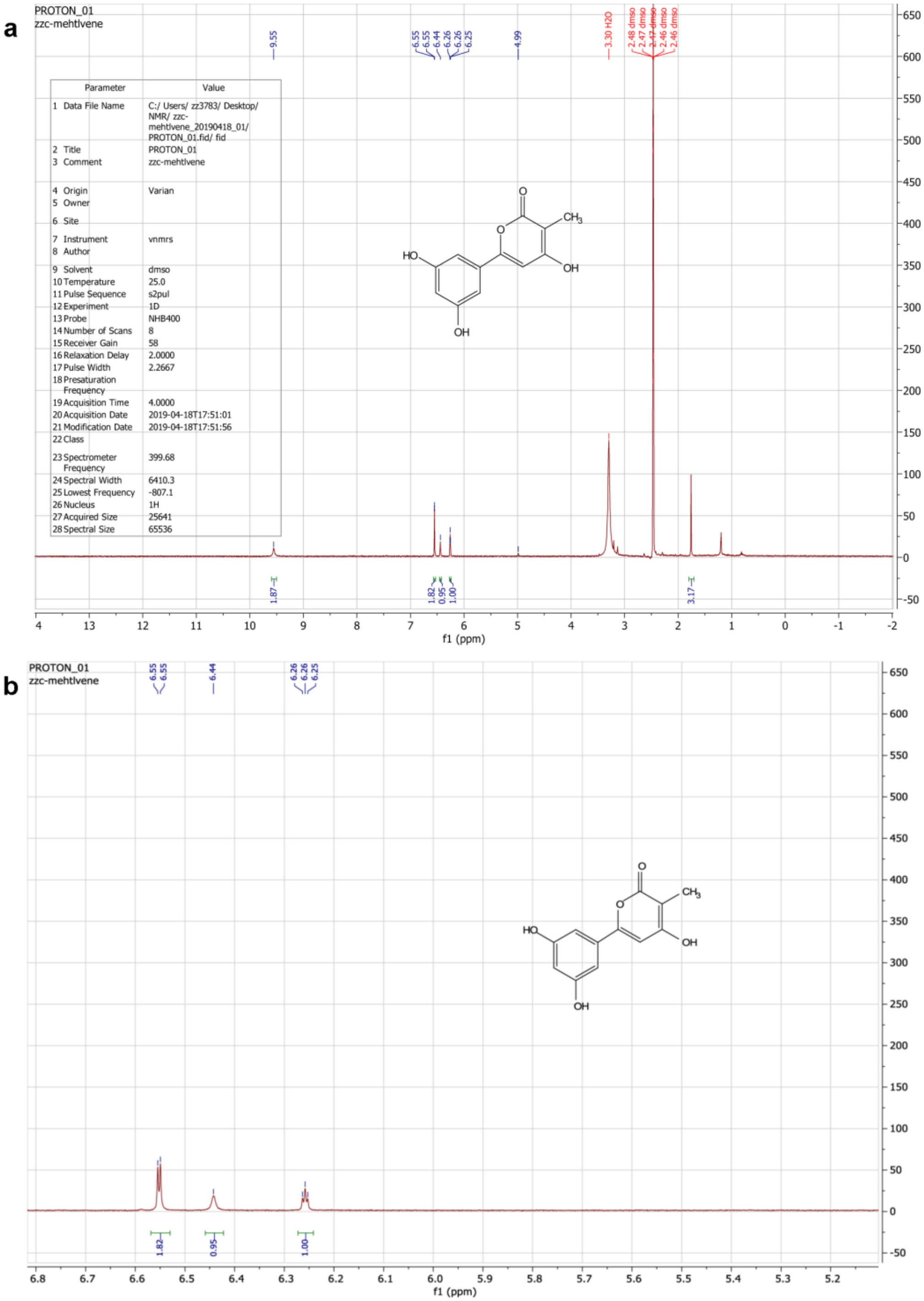

Supplementary Figure 13. ^{1}H NMR of methylvenemycin (3) in DMSO-d_6. a) Full spectrum. **b)** Expanded region.

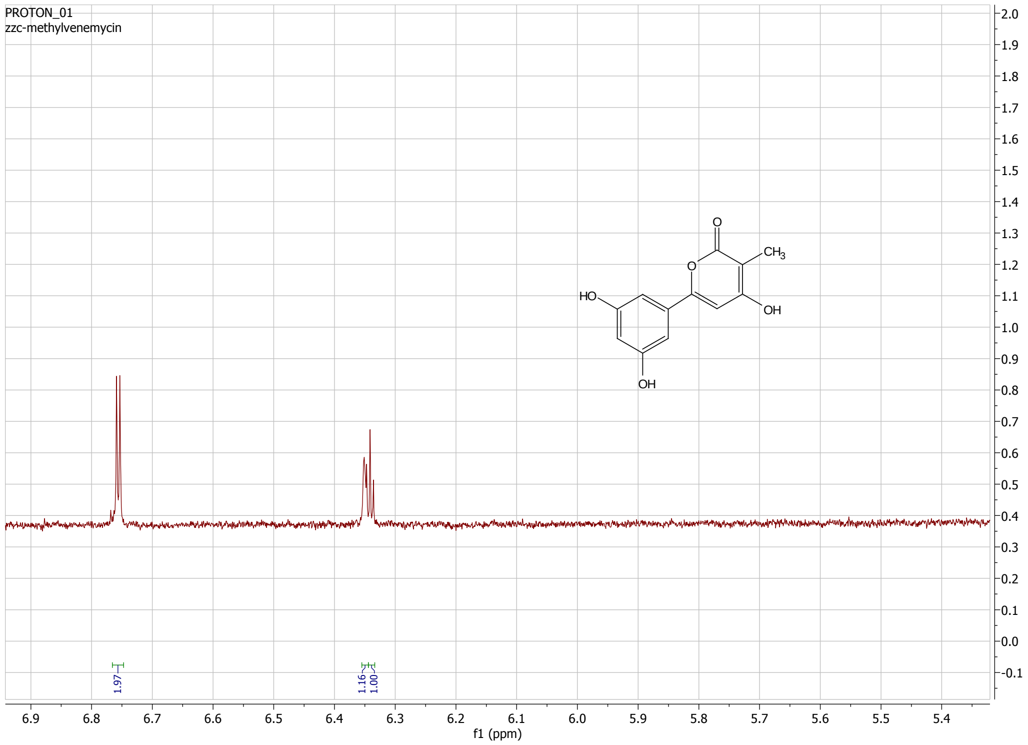

Supplementary Figure 14. ^{1}H NMR of methylvenemycin (3) in D_2O.

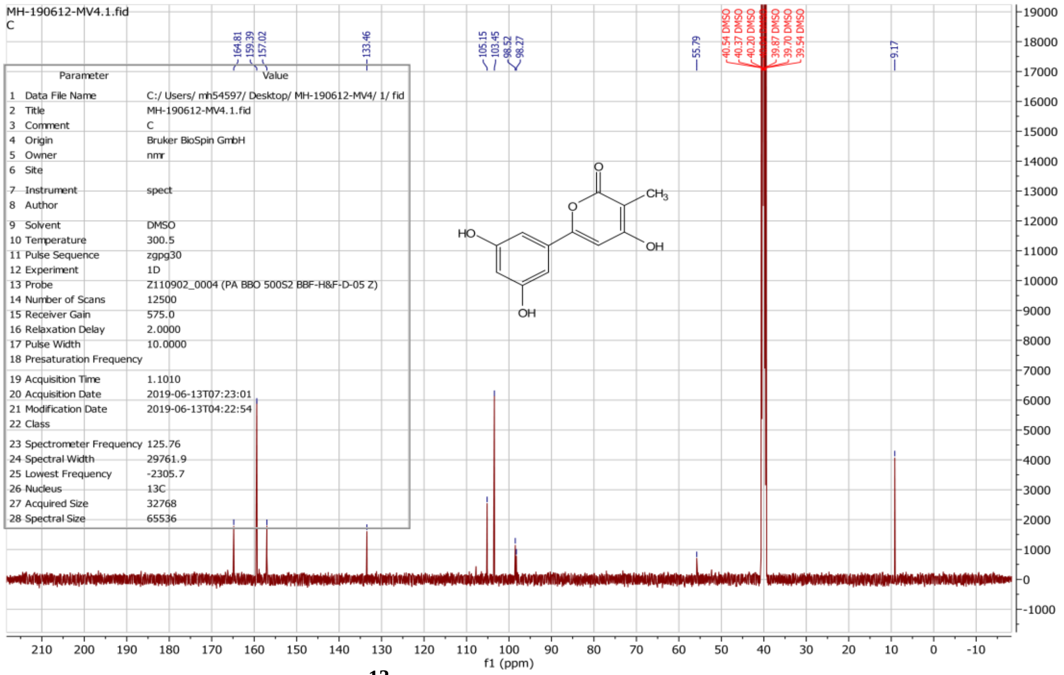

Supplementary Figure 15. ^{13}C NMR of methylvenemycin (3) in DMSO-d$_6$.

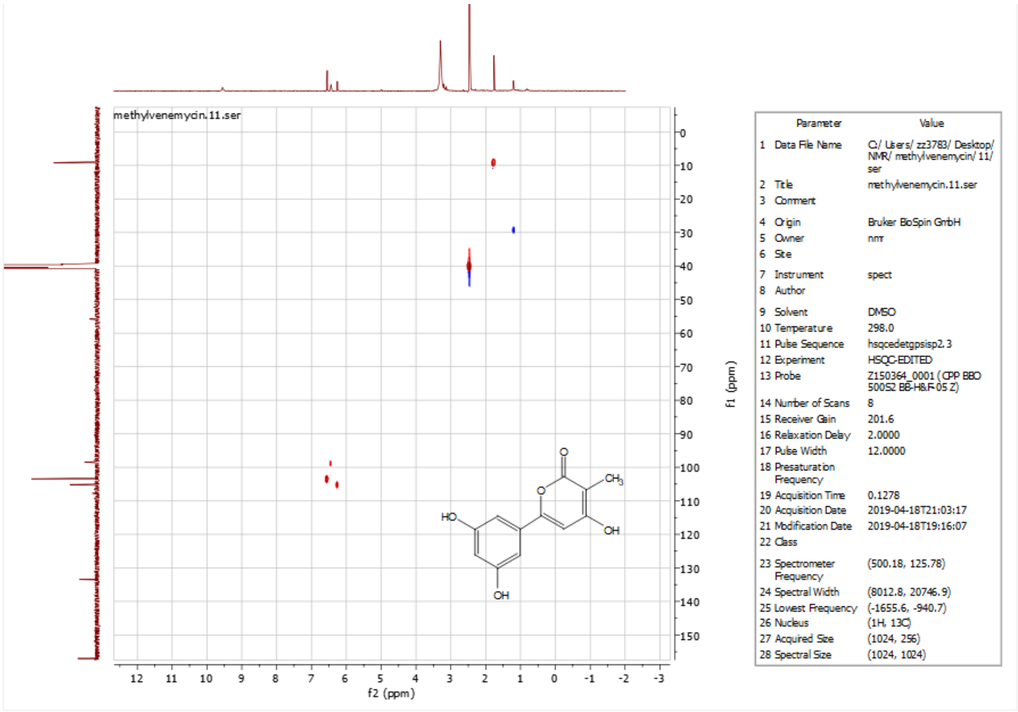

Supplementary Figure 16. ^{1}H-^{13}C-HSQC of methylvenemycin (3) in DMSO-d_6.

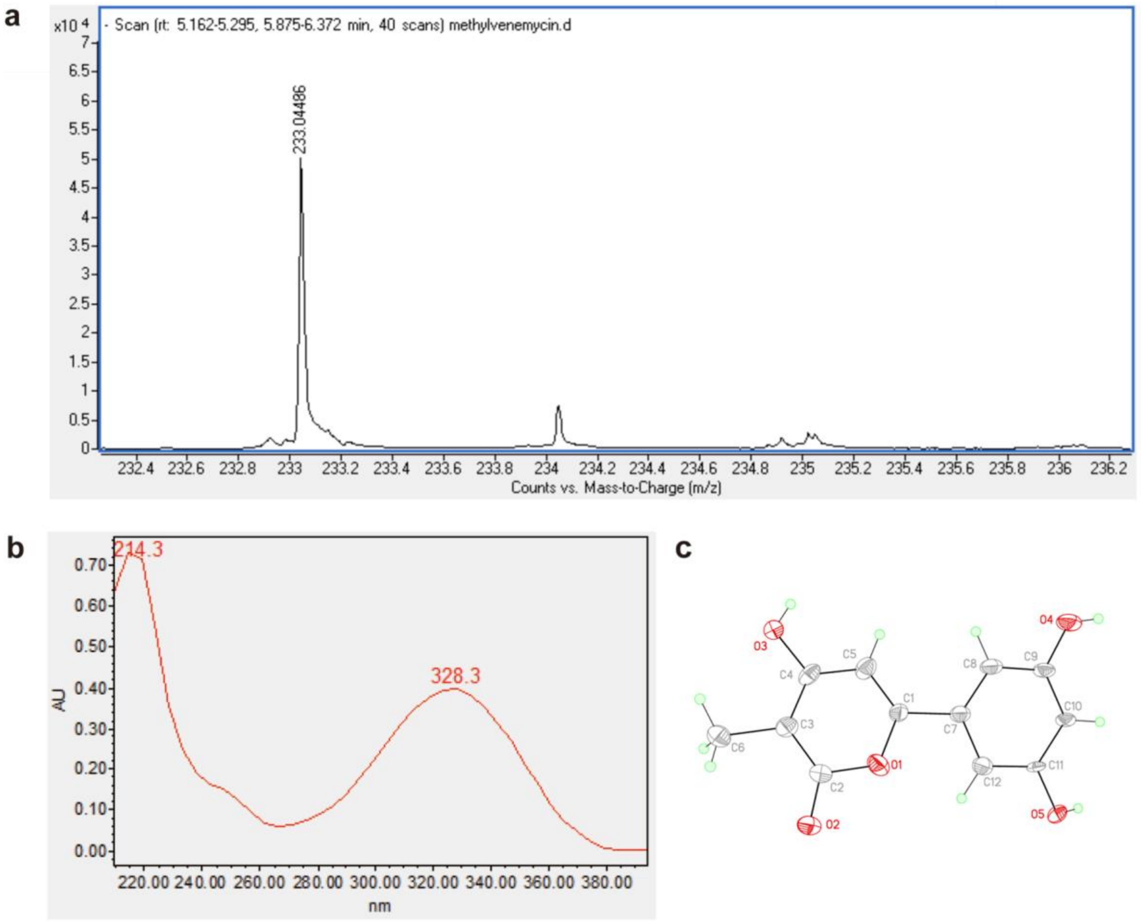

Supplementary Figure 17. Additional characterization of methylvenemycin (3). a) High resolution MS. 233.0455 *m*/*z* calculated, 233.0449 *m*/*z* found. **b)** Absorbance spectrum. **c)** X-ray crystal structure.

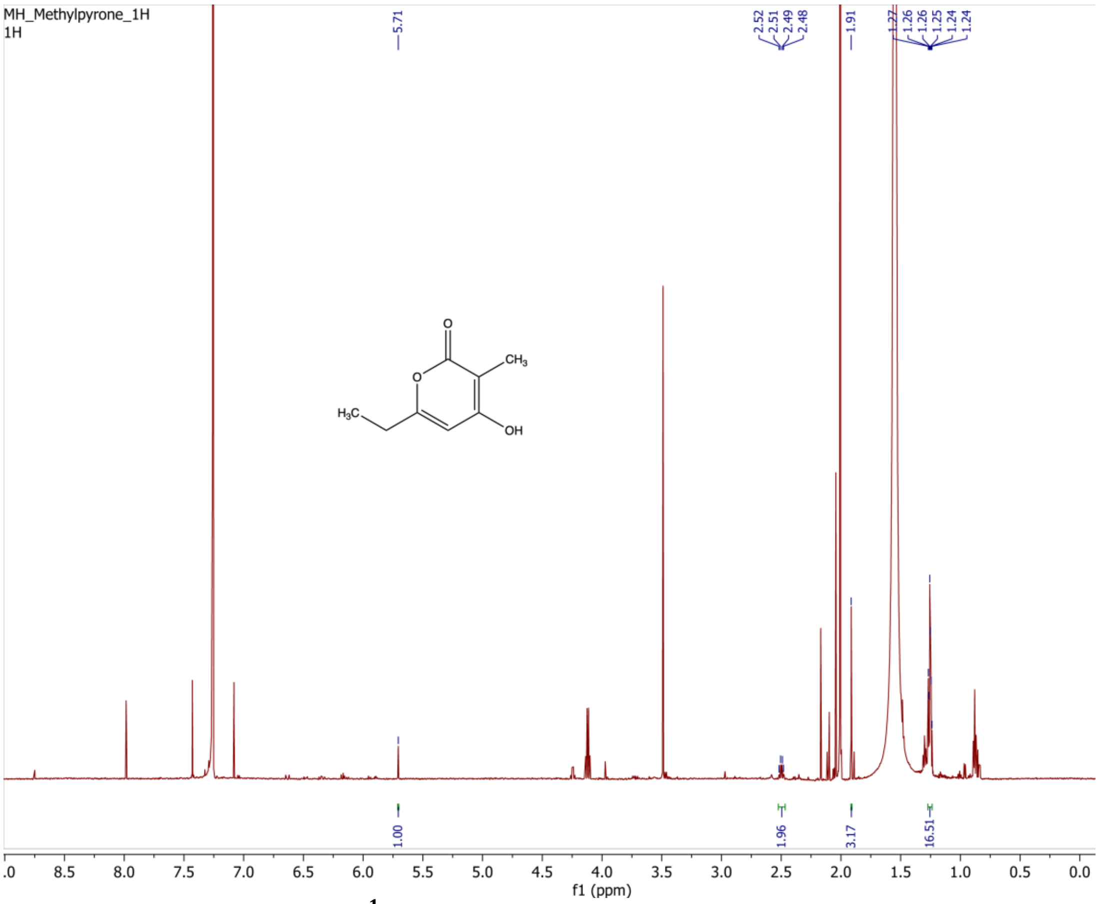

Supplementary Figure 18. ^{1}H NMR of methylpyrone (4) in $CDCl_3$.

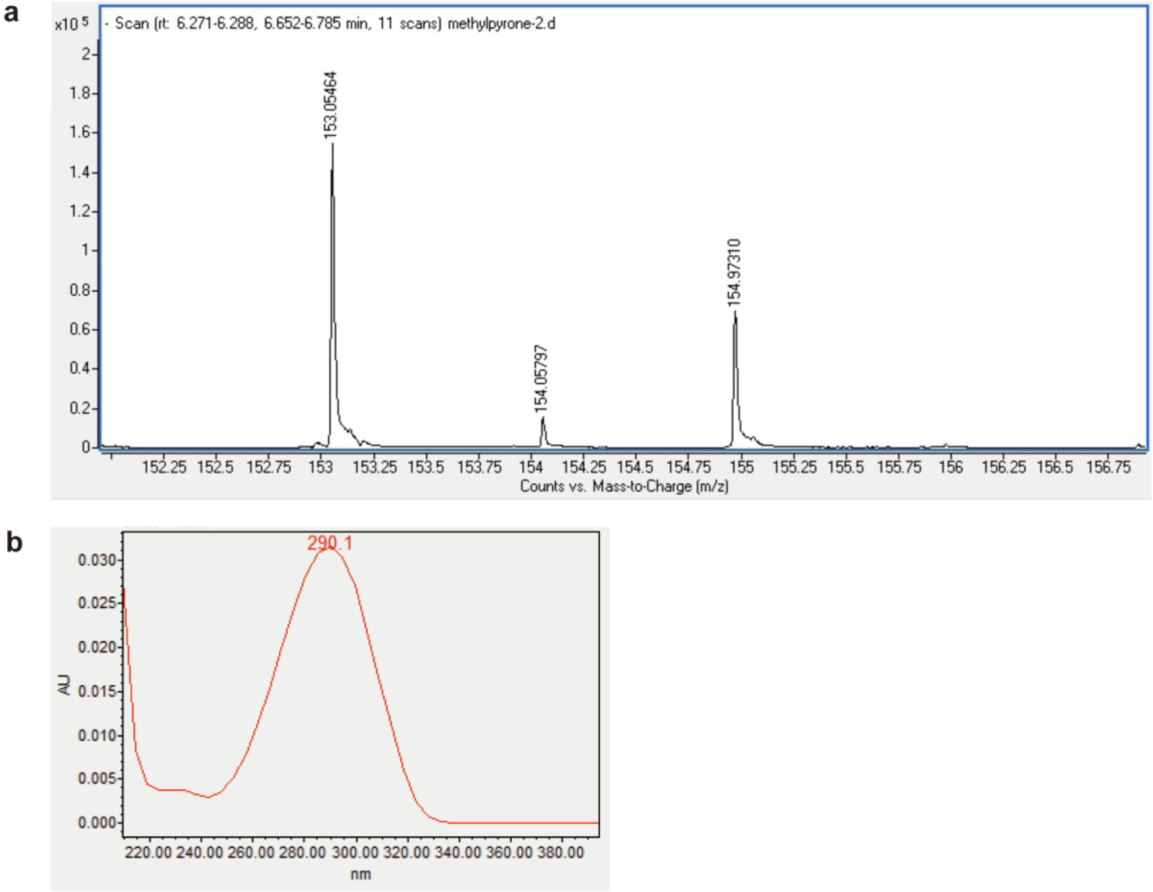

Supplementary Figure 19. Additional characterization of methylpyrone (4). a) High resolution MS. 153.0557 *m*/*z* calculated, 153.0546 *m*/*z* found. **b)** Absorbance spectrum.

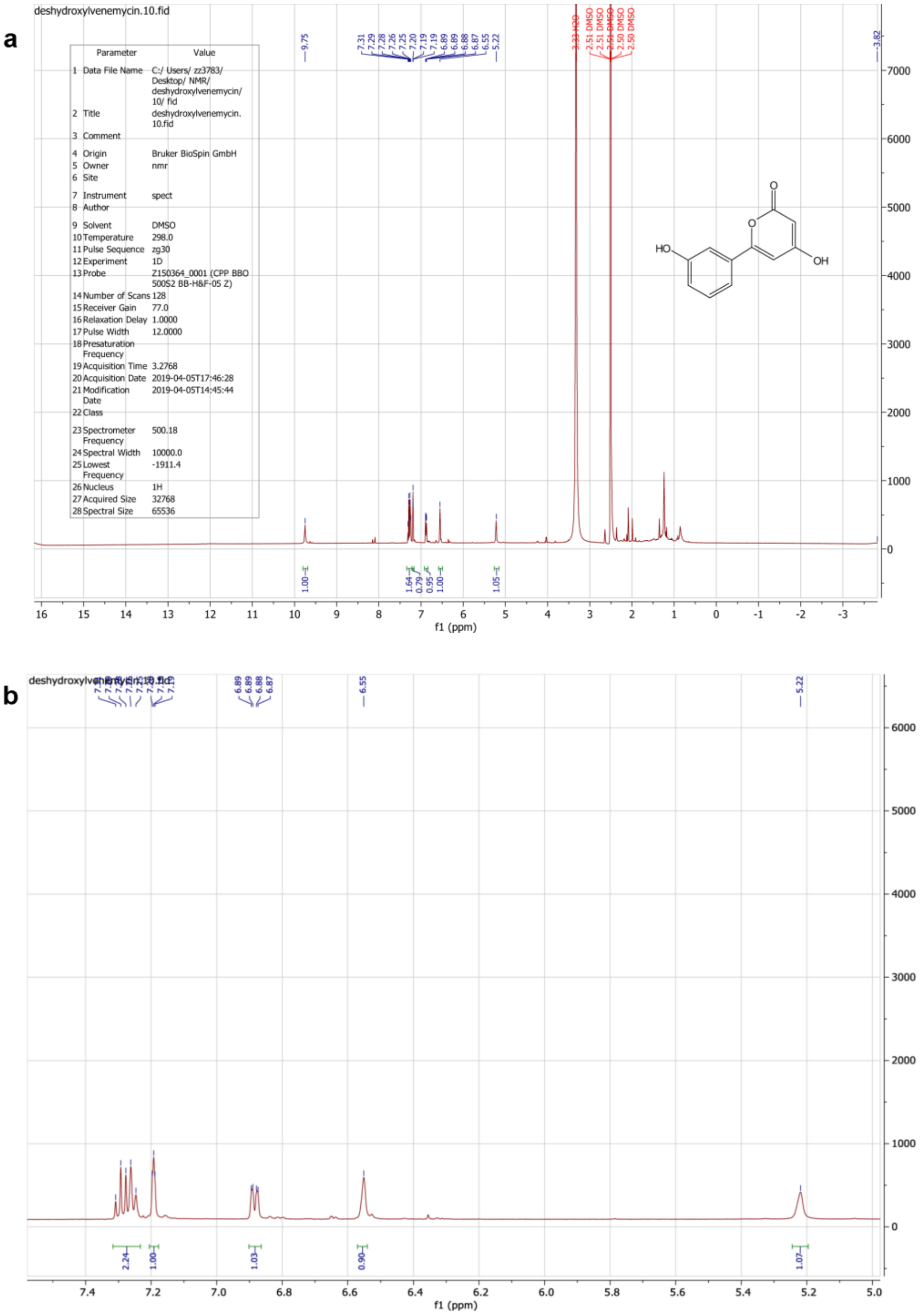

Supplementary Figure 20. ^{1}H NMR of deshydroxyvenemycin (5) in DMSO-d_6. a) Full spectrum. **b)** Expanded region.

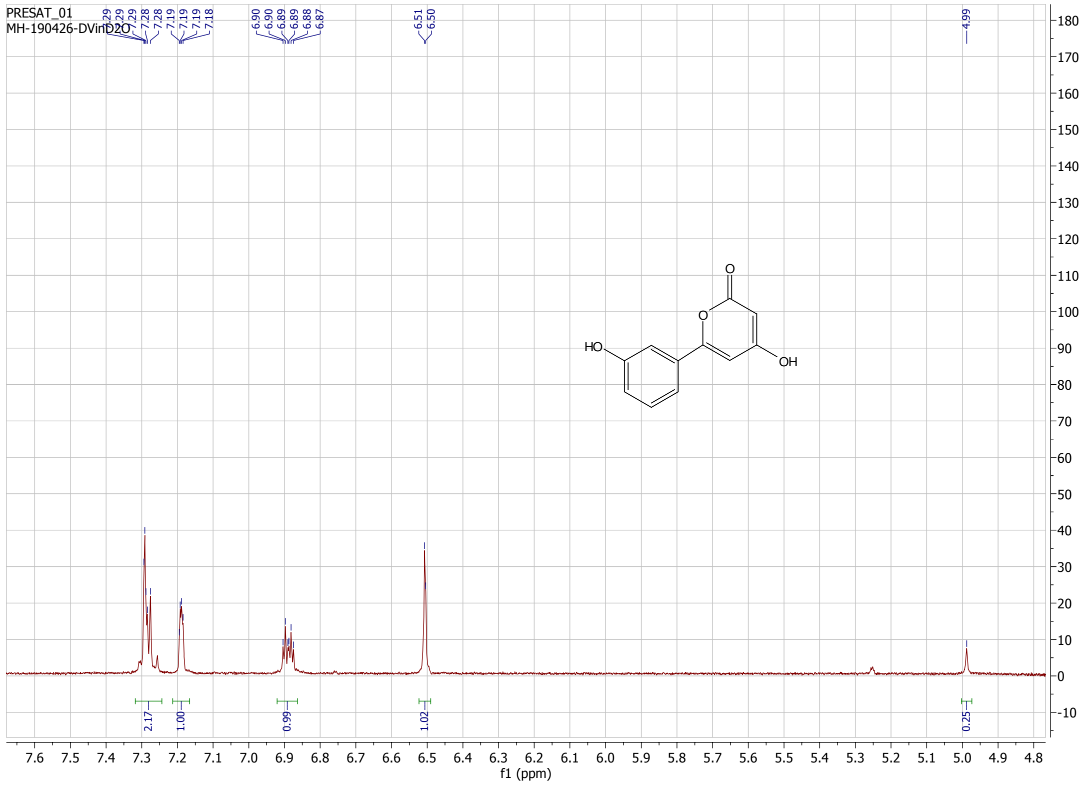

Supplementary Figure 21. ^{1}**H NMR of deshydroxyvenemycin (5) in** D_2O**.**

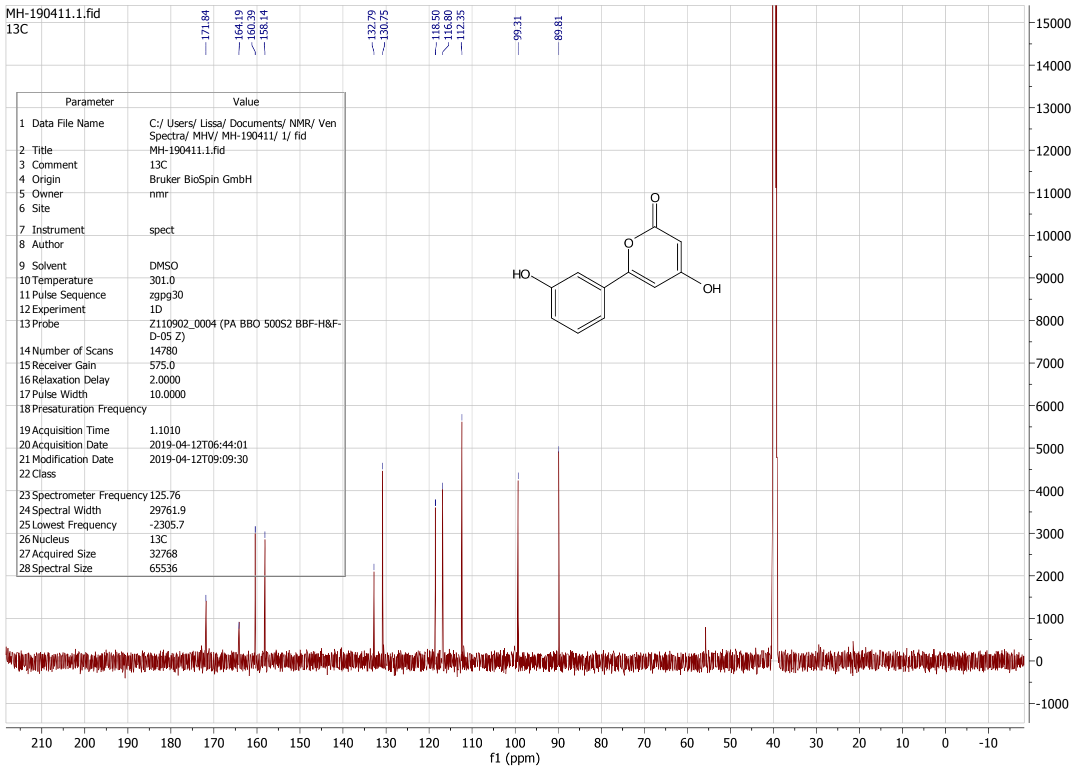

Supplementary Figure 22. ^{13}**C NMR of deshydroxyvenemycin (5) in DMSO-d$_6$.**

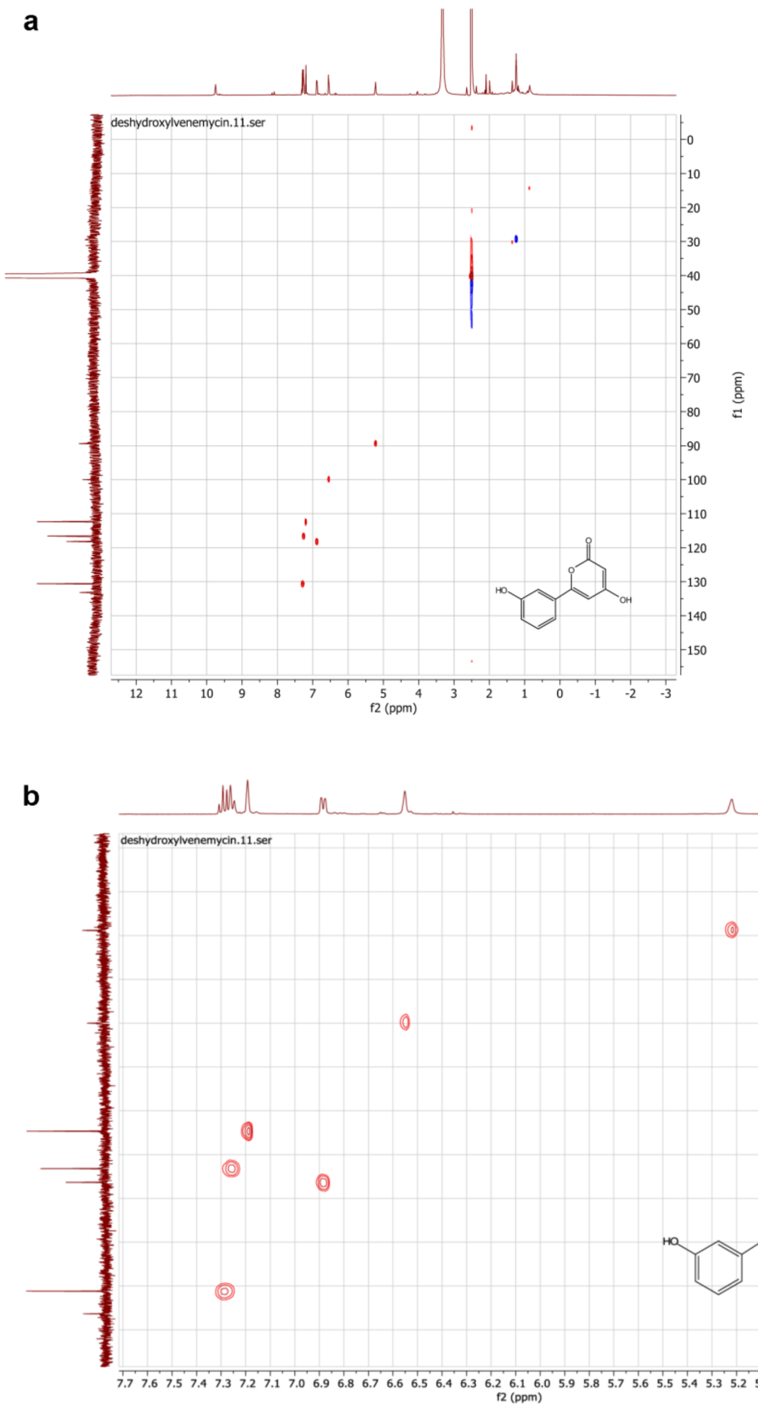

Parameter	Value
1 Data File Name	C:/ Users/ zz3783/ Desktop/ NMR/ deshydroxylvenemycin/ 11/ ser
2 Title	deshydroxylvenemycin.11.ser
3 Comment	
4 Origin	Bruker BioSpin GmbH
5 Owner	nmr
6 Site	
7 Instrument	spect
8 Author	
9 Solvent	DMSO
10 Temperature	298.0
11 Pulse Sequence	hsqcedetgpsisp2.3
12 Experiment	HSQC-EDITED
13 Probe	Z150364_0001 (CPP BBO 500S2 BB-H&F-05 Z)
14 Number of Scans	8
15 Receiver Gain	201.6
16 Relaxation Delay	2.0000
17 Pulse Width	12.0000
18 Presaturation Frequency	
19 Acquisition Time	0.1278
20 Acquisition Date	2019-04-05T17:48:51
21 Modification Date	2019-04-05T16:01:33
22 Class	
23 Spectrometer Frequency	(500.18, 125.78)
24 Spectral Width	(8012.8, 20746.9)
25 Lowest Frequency	(-1655.6, -940.7)
26 Nucleus	(1H, 13C)
27 Acquired Size	(1024, 256)
28 Spectral Size	(1024, 1024)

Supplementary Figure 23. ^{1}H-^{13}C-HSQC of deshydroxyvenemycin (5) in DMSO-d_6. **a)** Full spectrum. **b)** Expanded region.

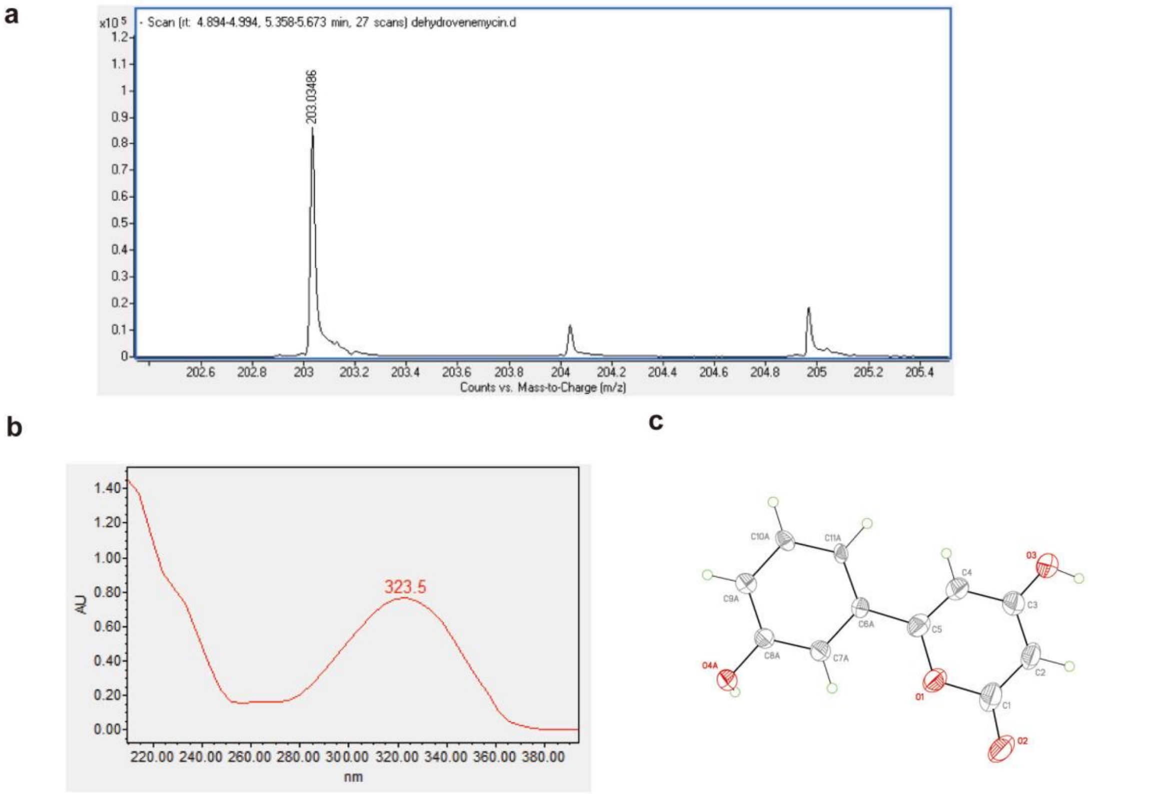

Supplementary Figure 24. Additional characterization of deshydroxyvenemycin (5). **a)** High resolution MS. 203.0350 *m*/*z* calculated, 203.0310 *m*/*z* found. **b)** Absorbance spectrum. **c)** X-ray crystal structure.

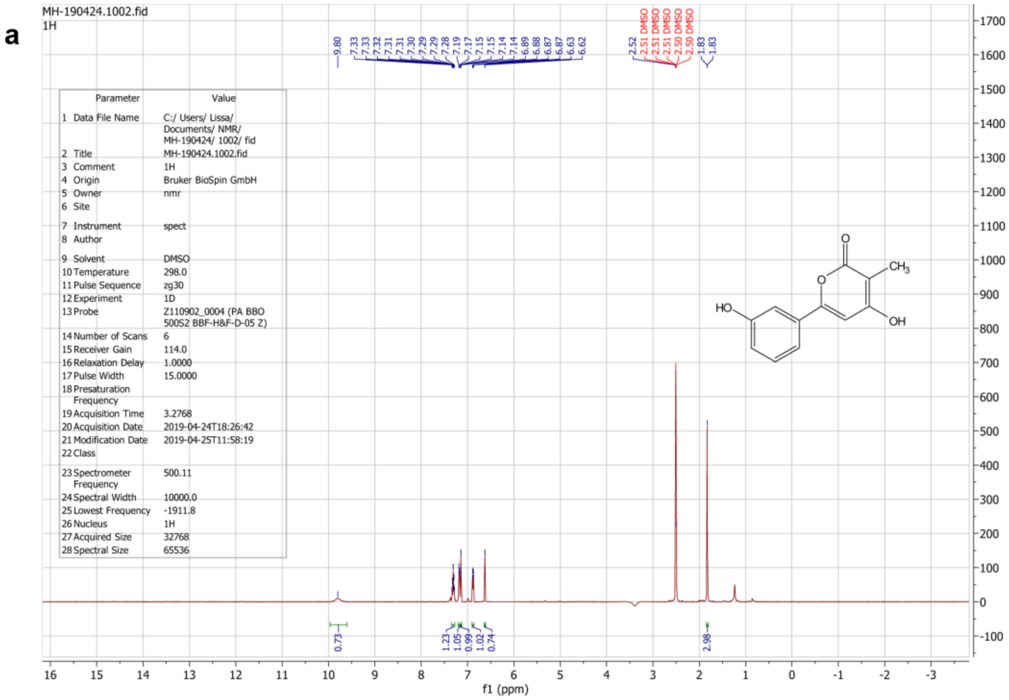

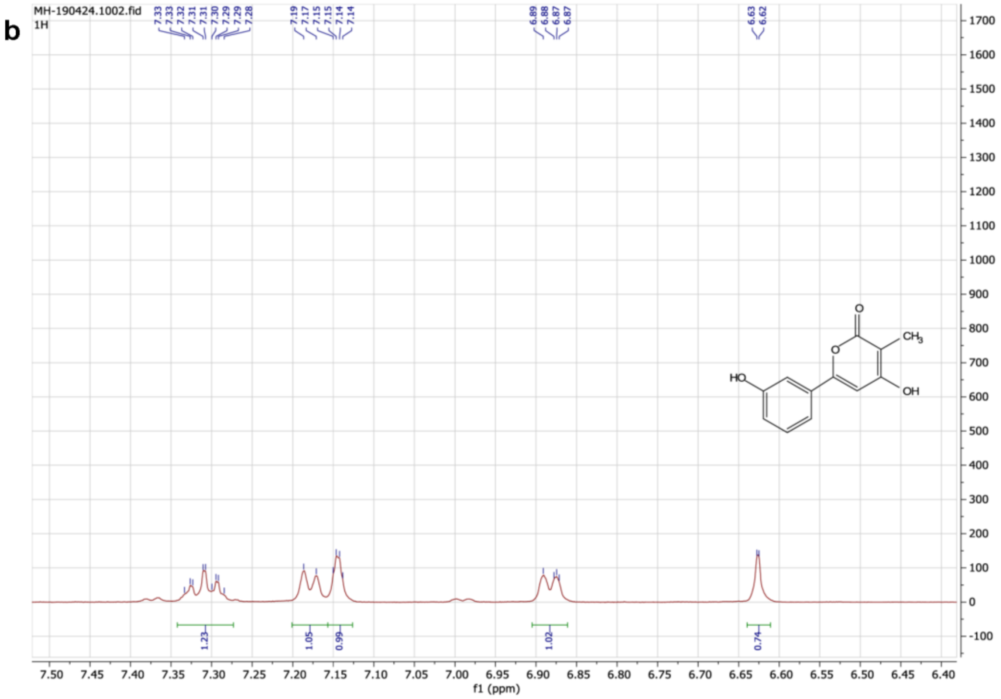

Supplementary Figure 25. ^{1}H NMR of deshydroxymethylvenemycin (6) in DMSO-d_6. **a)** Full spectrum. **b)** Expanded region.

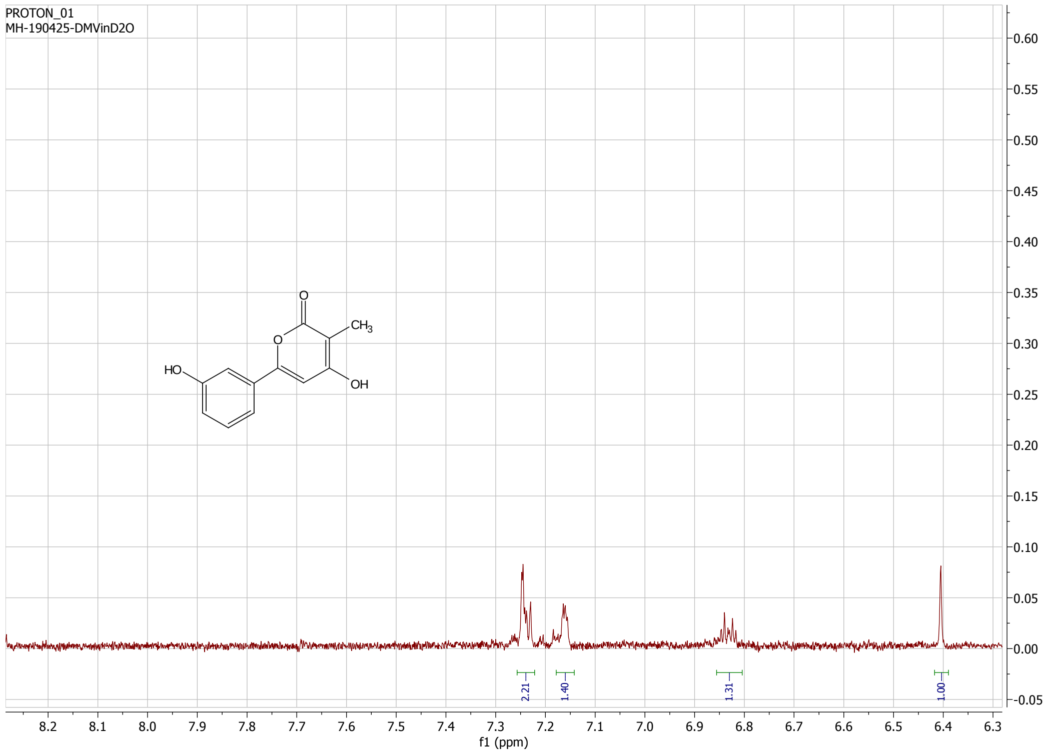

Supplementary Figure 26. ^{1}H NMR of deshydroxymethylvenemycin (6) in D_2O.

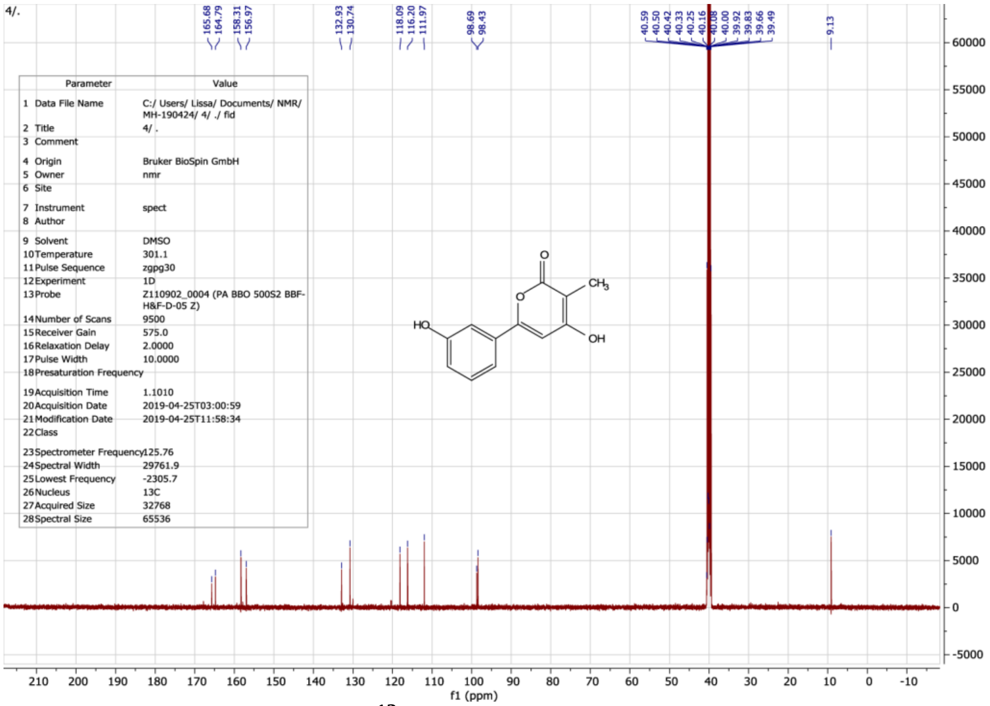

Supplementary Figure 27. ^{13}**C NMR of deshydroxymethylvenemycin (6) in DMSO-d$_6$.**

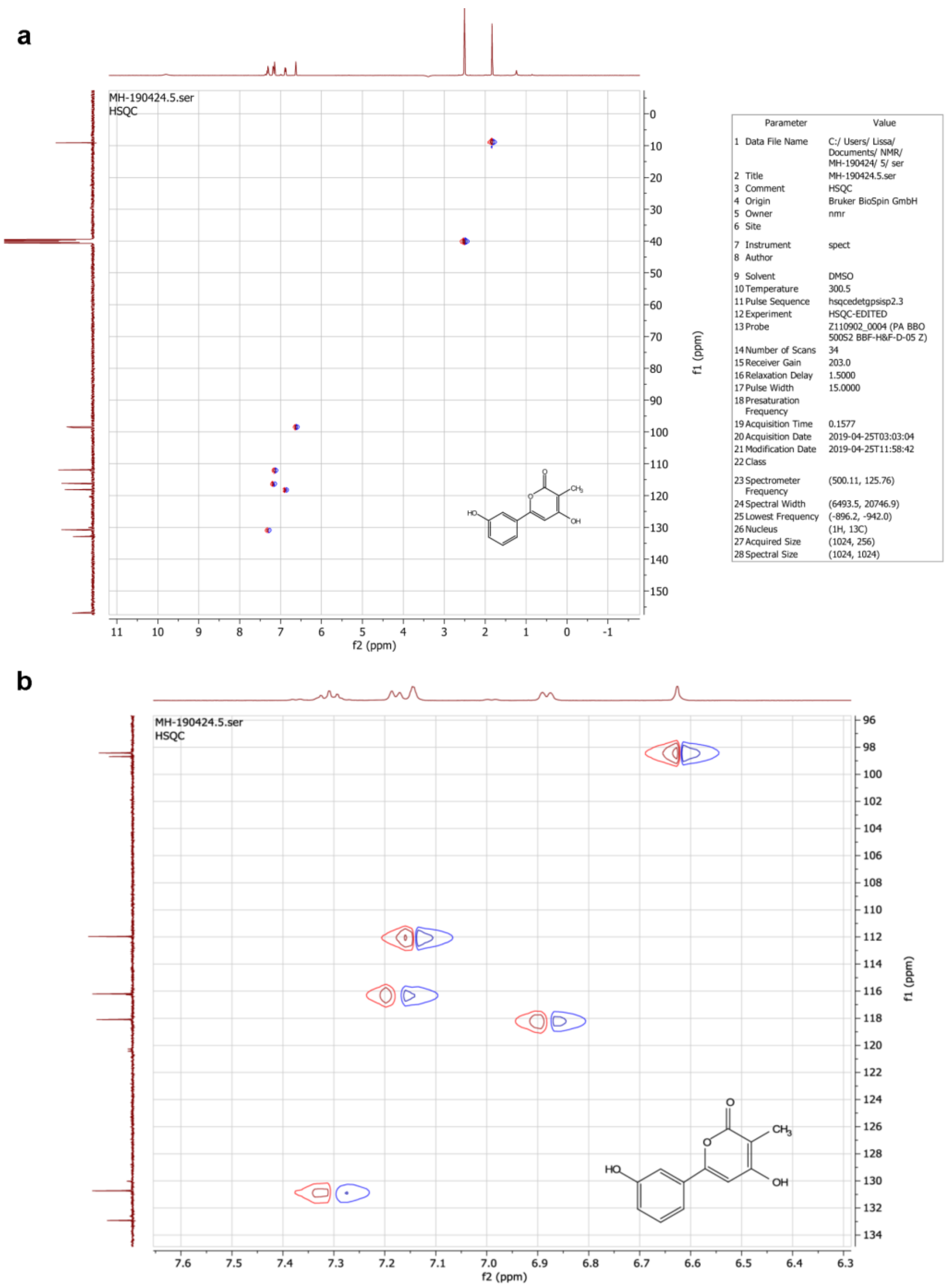

Supplementary Figure 28. ^{1}H-^{13}C-HSQC of deshydroxymethylvenemycin (6) in DMSO-d_6. a) Full spectrum. **b)** Expanded region.

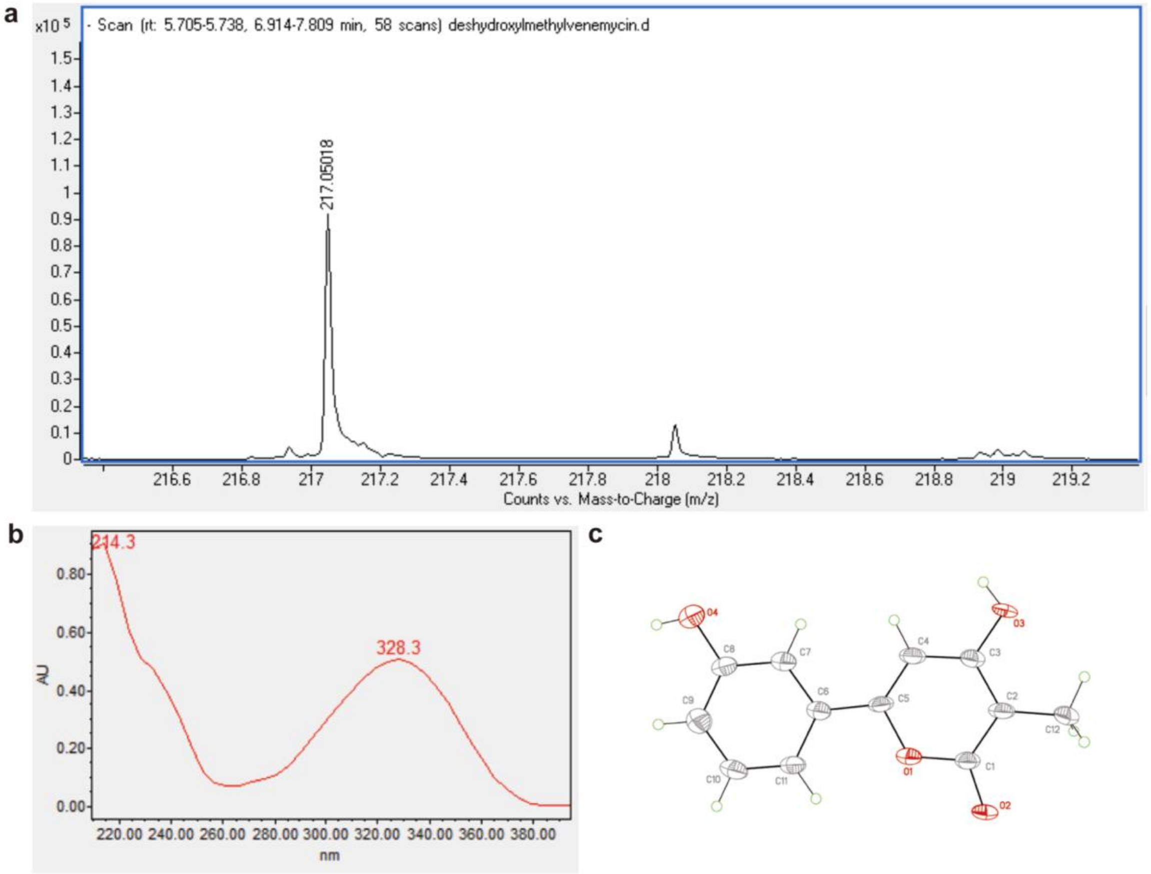

Supplementary Figure 29. Additional characterization of deshydroxymethylvenemycin (6). a) High resolution MS. 217.0506 *m/z* calculated, 217.0502 *m/z* found. **b)** Absorbance spectrum. **c)** X-ray crystal structure.

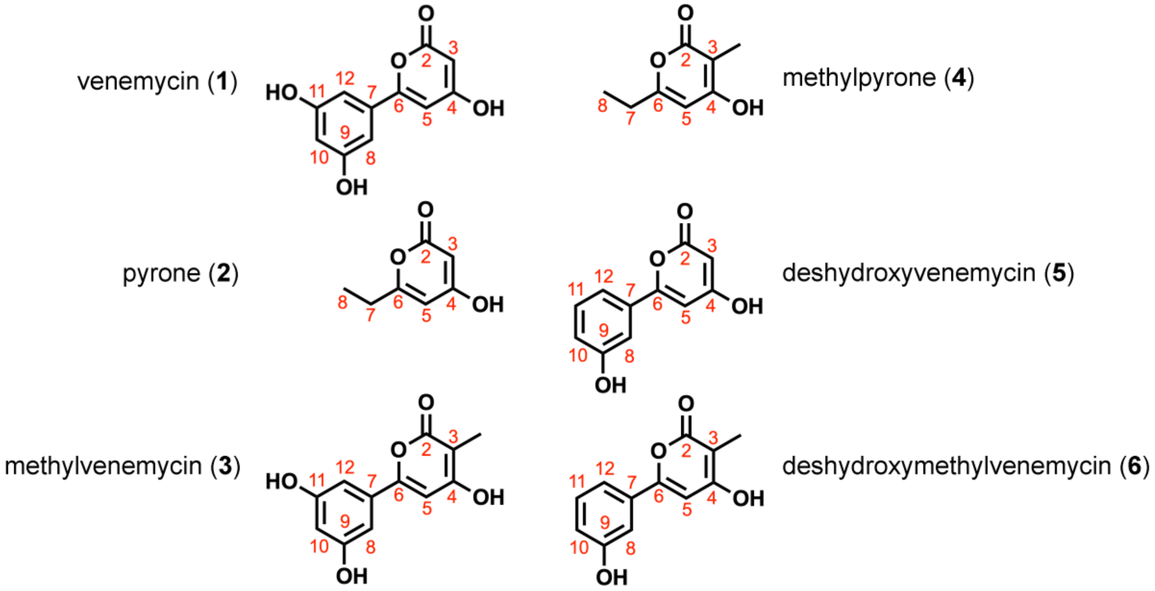

Supplementary Figure 30. Numbering system for polyketide products.

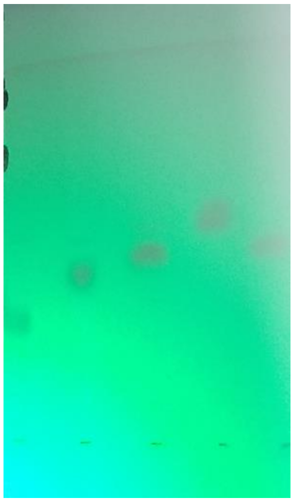

Supplementary Figure 31. TLC of compounds produced by combinatorial biosynthesis. Left to right: venemycin (**1**), methylvenemycin (**3**), deshydroxyvenemycin (**5**), deshydroxymethylvenemycin (**6**), and pyrone (**2**). Run in EtOAc:hexanes:HCOOH, 66:33:1, viewed under 254 nm light.

Supplementary Table 1. Primers used in this study.

VemG F	tgccgcgcggcagccatatgGTGACGCAAGTCGATGACATCCGGCCCCTA
VemG R	tggtggtggtggtgctcgagTCAGGGTCCCTTCAGTTCGTTGTCGATGA
VemH F	aagaaggagatatacatatgACGGGCACCGAGGAGAAGCTGGT
VemH R	tggtggtggtggtgctcgagCAGCAGTGACCGTGACCACTGCTCGA
PikAI updated F	tggtgccgcgcggcagccatATGTCTTCAGCCGGAATTACCAGGACCGGT
PikAI updated R	ggcggcagggcgggcggcgcGACAACCACCGGGGCCTCTTCGAGCA
PikAI traditional F	tggtgccgcgcggcagccatATGTCTTCAGCCGGAATTACCAGGACCGGT
PikAI traditional R	cggcggccggtgccgtcgacGACCCGGTCGTCGTCATCGGCATGAGC
VemG AT-ACP-^{c}DD F	gcgccgcccgccctgccgcc
VemG AT-ACP-^{c}DD R	tggtggtggtggtgctcgagTCAGGGTCCCTTCAGTTCGTTGTCGATGA
VemG KS-AT-ACP-^{c}DD F	gacccggtcgtcgtcatcggCATGAGCT
VemG KS-AT-ACP-^{c}DD R	tggtggtggtggtgctcgagTCAGGGTCCCTTCAGTTCGTTGTCGATGA
PikAIV updated F	gccccgaggcgcggtcgcggGTCGAGCCGCCGGCCGGTGGCGGCGT
PikAIV updated R	tggtggtggtggtgctcgagCTTGCCCGCCCCCTCGATGCCCTCGAT
PikAIV traditional F	tttcaagcggatccctcagcATTCTTTTCAACATCA
PikAIV traditional R	tggtggtggtggtgctcgagCTTGCCCGCCCCCTCGATGCCCTCGAT
VemH ^{N}DD-KS F	aagaaggagatatacatatgACGGGCACCGAGGAGAAGCTGGT
VemH ^{N}DD-KS R	gggctcctcgggtggggcc
VemH ^{N}DD F	aagaaggagatatacatatgACGGGCACCGAGGAGAAGCTGGT
VemH ^{N}DD R	cggctcgccgccagcgcgcCCCGCAACT
pET28b-His10 F	agccatcatcatcatcatcaTCATCATCATCACAGCAG
pET28b-His10 R	tgatgatgatgatgatggctGCTGCCCATGGTATATCTCC

Note: Lowercase indicates SLiCE overlapping sequence.

Supplementary References

1 Sheldrick, G. M. SHELXT. A program for crystal structure solution. *Acta Cryst* **A71**, 3-8 (2015).
2 Sheldrick, G. M. SHELXL-2016/6. Program for the refinement of crystal structures. *Acta Cryst.*, **C71**, 9-18 (2015).
3 Spek, A. L. PLATON, a multipurpose crystallographic tool. *Acta Cryst* **D65**, 148-155 (2009).
4 Dolomanov, O. V., Bourhis, L. J., Gildea, R. J., Howard, J. A. K. & Puschmann, H. OLEX2: a complete structure solution, refinement and analysis program. *J Appl Cryst* **42**, 339-341 (2009).
5 Farrugia, L. J. WinGX 1.64. An integrated system of windows programs for the solution, refinement and analysis of single crystal x-ray diffraction data. *J Appl Cryst* **32**, 837-838 (1999).
6 International Tables for X-ray Crystallography, Vol. C (1992), A. J. C. Wilson, editor, Boston: Kluwer Academic Press.
