# Supplementary material for: An in vitro platform for engineering and harnessing modular polyketide synthases
Source: Nat Commun. 2020 Jan 3;11:80. doi: 10.1038/s41467-019-13811-0 (PMC6941969; doi:10.1038/s41467-019-13811-0)
Supplement: Supplementary file 3 — Description of Additional Supplementary Files [file 41467_2019_13811_MOESM3_ESM.docx]

**Description of Additional Supplementary Files**

File Name: Supplementary Data 1
Description: Hybrid assembly line sequences and fusion sites
